# Brain MRI detects early-stage alterations and disease progression in Friedreich ataxia

**DOI:** 10.1093/braincomms/fcad196

**Published:** 2023-07-06

**Authors:** Isaac M Adanyeguh, James M Joers, Dinesh K Deelchand, Diane H Hutter, Lynn E Eberly, Bin Guo, Isabelle Iltis, Khalaf O Bushara, Pierre-Gilles Henry, Christophe Lenglet

**Affiliations:** Center for Magnetic Resonance Research and Department of Radiology, University of Minnesota Medical School, Minneapolis, MN 55455, USA; Center for Magnetic Resonance Research and Department of Radiology, University of Minnesota Medical School, Minneapolis, MN 55455, USA; Center for Magnetic Resonance Research and Department of Radiology, University of Minnesota Medical School, Minneapolis, MN 55455, USA; Center for Magnetic Resonance Research and Department of Radiology, University of Minnesota Medical School, Minneapolis, MN 55455, USA; Division of Biostatistics, School of Public Health, University of Minnesota, Minneapolis, MN 55455, USA; Division of Biostatistics, School of Public Health, University of Minnesota, Minneapolis, MN 55455, USA; Center for Magnetic Resonance Research and Department of Radiology, University of Minnesota Medical School, Minneapolis, MN 55455, USA; Department of Neurology, University of Minnesota Medical School, Minneapolis, MN 55455, USA; Center for Magnetic Resonance Research and Department of Radiology, University of Minnesota Medical School, Minneapolis, MN 55455, USA; Center for Magnetic Resonance Research and Department of Radiology, University of Minnesota Medical School, Minneapolis, MN 55455, USA

**Keywords:** magnetic resonance imaging, diffusion imaging, fixel-based analysis, imaging biomarkers, longitudinal imaging

## Abstract

Friedreich ataxia is a progressive neurodegenerative disorder characterized by cerebellar and spinal atrophy. However, studies to elucidate the longitudinal progression of the pathology in the brain are somewhat inconsistent and limited, especially for early-stage Friedreich ataxia. Using a multimodal neuroimaging protocol, combined with advanced analysis methods, we sought to identify macrostructural and microstructural alterations in the brain of patients with early-stage Friedreich ataxia to better understand its distribution patterns and progression. We enrolled 28 patients with Friedreich ataxia and 20 age- and gender-matched controls. Longitudinal clinical and imaging data were collected in the patients at baseline, 12, 24 and 36 months. Macrostructural differences were observed in patients with Friedreich ataxia, compared to controls, including lower volume of the cerebellar white matter (but not cerebellar grey matter), superior cerebellar peduncle, thalamus and brainstem structures, and higher volume of the fourth ventricle. Diffusion tensor imaging and fixel-based analysis metrics also showed microstructural differences in several brain regions, especially in the cerebellum and corticospinal tract. Over time, many of these macrostructural and microstructural alterations progressed, especially cerebellar grey and white matter volumes, and microstructure of the superior cerebellar peduncle, posterior limb of the internal capsule and superior corona radiata. In addition, linear regressions showed significant associations between many of those imaging metrics and clinical scales. This study provides evidence of early-stage macrostructural and microstructural alterations and of progression over time in the brain in Friedreich ataxia. Moreover, it allows to non-invasively map such brain alterations over a longer period (3 years) than any previous study, and identifies several brain regions with significant involvement in the disease progression besides the cerebellum. We show that fixel-based analysis of diffusion MRI data is particularly sensitive to longitudinal change in the cerebellar peduncles, as well as motor and sensory white matter tracts. In combination with other morphometric measures, they may therefore provide sensitive imaging biomarkers of disease progression for clinical trials.

## Teaser

Adanyeguh *et al*. sought to evaluate the brain longitudinal alterations in an early-stage Friedreich Ataxia cohort. Unlike existing studies performed over shorter durations, Adanyeguh *et al*. mapped cerebral and cerebellar alterations over three years that showed high effect sizes and correlated with clinical scores.

## Graphical Abstract

**Graphical Abstract:**
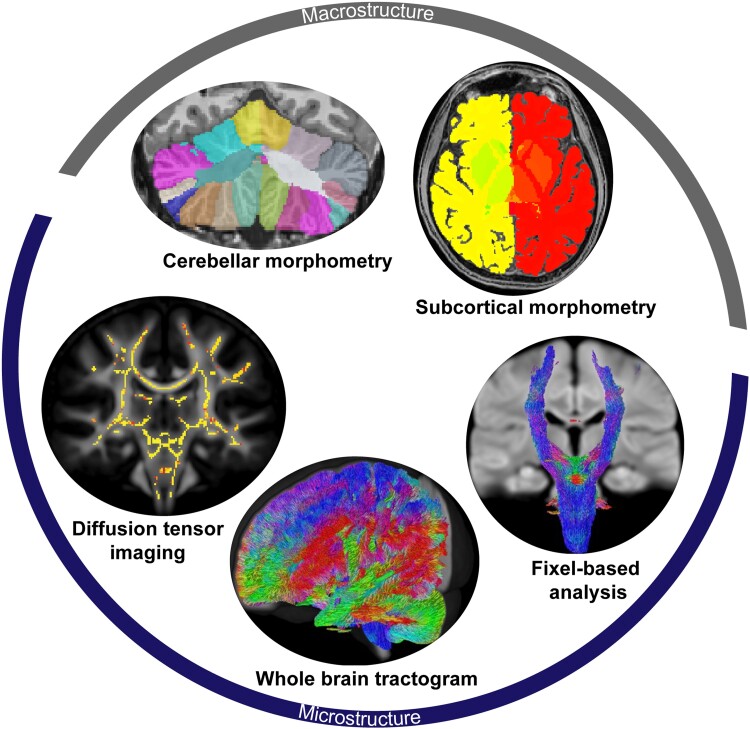

## Introduction

Friedreich ataxia (FRDA) is an inherited autosomal recessive neurodegenerative disorder characterized by progressive limb and gait ataxia, dysarthria, cardiomyopathy and skeletal deformities.^1,2^ The disease typically leads to wheelchair-dependency in ∼10 years,^2^ and there is currently no approved disease-modifying treatment.

Advances in neuroimaging have allowed *in vivo* evaluation of neurodegenerative disorders with some imaging biomarkers showing higher effect sizes compared to clinical scales,^3,4^ therefore potentially requiring fewer patients in clinical trials. Several cross-sectional studies using magnetic resonance imaging (MRI) have shown brain atrophy in the cerebellum and brainstem of patients with FRDA.^5-12^ Atrophy in the cerebral hemispheres has also been reported.^7,11^ Furthermore, diffusion MRI (dMRI) has been used to identify microstructural alterations in the cerebral and cerebellar regions.^6,8,10,11,13-19^ It has also been used to investigate degeneration of the spinal cord (see our previous work^20^ and references therein).

While it is important to study these macro and microstructural differences between individuals with FRDA and controls, it is also essential to understand how these alterations progress over time in the patient population. However, very few longitudinal brain imaging studies have been reported in FRDA,^8,11,21,22^ and reported findings have not been entirely consistent: while no longitudinal morphometric changes have been reported in some studies,^8,21^ a recent study^11^ detected a higher rate of volume reduction in the superior cerebellar peduncles (SCPs), right peri-thalamic and posterior cerebral regions in individuals with FRDA than in controls. Furthermore, while this recent study did not observe longitudinal microstructural alterations,^11^ other previous studies have reported such longitudinal microstructural alterations in the cerebral white matter, corpus callosum, pyramidal tracts and superior cerebellar peduncle brain regions.^8,21^

One way to improve the sensitivity of dMRI measures is to use more advanced data acquisition and analysis techniques. Among the several microstructural cross-sectional studies in FRDA, only a few^17,23^ used higher order diffusion models to try and overcome the inability of diffusion tensor imaging (DTI) to account for crossing-fibres, which are present in ∼90% of the brain white matter.^24^ Likewise, no study has so far analysed individual fibre populations per voxel, known as fixel, to estimate their corresponding fibre density (FD, i.e. intra-axonal volume fraction), fibre bundle cross-section (FC, i.e. total area occupied by the axons) and a combination of FD and FC (FDC, i.e. fibre density and cross-section).^25^ This technique, known as fixel-based analysis (FBA), has been shown to provide robust microstructural measures with high effect sizes^3,4^ in other neurodegenerative disorders, which motivated its application to FRDA here.

The present study thus aimed to explore the macrostructural and microstructural alterations in patients with early-stage FRDA (defined here as functional staging ≤ 3) using conventional morphometric and DTI metrics, as well as the more advanced FBA technique to evaluate fibre density and cross-section in FRDA. We hypothesized that FBA might be more sensitive to alterations and longitudinal changes than DTI as it explicitly models complex white matter (e.g. crossing) fibre configurations via fibre orientation distributions (FODs), and enables the estimation of fibre-specific microstructure metrics (e.g. density). We first sought to identify cross-sectional differences between individuals with FRDA and matched controls. Second, we sought to determine longitudinal changes of these measures in individuals with FRDA.

## Materials and methods

### Participants

The University of Minnesota Institutional Review Board approved the study, which was conducted in accordance with the Declaration of Helsinki. Adult participants signed a written informed consent. Parents of minor participants signed the informed consent, and the minor participant signed an informed assent. FRDA participants were recruited after a confirmed FRDA diagnosis. Subjects were excluded if they presented other neurological disease. Other exclusion criteria were pregnancy, claustrophobia, restless leg syndrome, diabetes, smoking and history of alcoholism. A 3 week withholding of antioxidants and a 36 h withholding of benzodiazepine (and other ataxia-specific medication) were requested prior to scanning.

Twenty-eight individuals at an early stage of FRDA (14 males and 14 females; mean age: 19.0 ± 7.3 years; time from diagnosis: 2.3 ± 2.6 years; disease duration: 5.5 ± 4.0 years) and 20 healthy controls (11 males and 9 females; mean age: 20.4 ± 7.1 years) were recruited for the study (Table 1) as previously described.^20^ Some individuals with FRDA did not return for follow-up visits due to several reasons including: too progressed (*n* = 1), rod placement (*n* = 2), brain arteriovenous anomaly (*n* = 1), claustrophobia (*n* = 1) and no longer interested or too busy (*n* = 4).^20^ Furthermore, five additional healthy volunteers (not included in the 20 controls) were scanned before and after scanner upgrade (see below).

**Table 1:** Cohort demographics and clinical characteristics

	Cross-sectional cohort	
	Control	FRDA
Number of subjects	20 (11M/9F)	28 (14M/14F)
Age (years)	20.4 (7.1)	19.0 (7.3)
Age of onset (years)	-	13.4 (5.2)
Disease duration (years)	-	5.5 (4.0)
Time from diagnosis (years)	-	2.3 (2.6)
GAA repeats (shorter)^a^	-	597 (179)
GAA repeats (longer)	-	959 (205)
SARA (0–40)^b^	0 (0)	8.9 (2.8)
FARS total neuro (0–117)	0 (0)	42.7 (13.6)
Functional staging (0–6)	0 (0)	2.0 (1.0)
ADL (0–36)	0 (0)	5.8 (3.5)
9HPT dominant (s)	19.2 (2.8)	40.4 (12.0)
9HPT non-dominant (s)	19.2 (2.4)	43.4 (13.0)
Timed walk (s)^c^	4.3 (0.6)	9.4 (19.1)

	Longitudinal cohort (FRDA only)			
	Baseline	1-year follow-up	2-year follow-up	3-year follow-up
Number of subjects	21 (10M/11F)	21 (10M/11F)	19 (9M/10F)	14 (5M/9F)
Age (years)	19.0 (7.7)	20.1 (7.7)	21.5 (8.0)	24.5 (8.9)
Age of onset (years)	13.8 (5.8)	13.8 (5.8)	14.1 (6.0)	15.4 (5.5)
Disease duration (years)	5.1 (3.9)	6.3 (3.9)	7.4 (4.2)	9.1 (5.2)
Time from diagnosis (years)	2.1 (2.9)	3.2 (2.9)	4.2 (3.0)	5.6 (3.7)
GAA repeats (shorter)^a^	598 (191)	598 (191)	596 (200)	560.4 (170.2)
GAA repeats (longer)	987 (221)	987 (221)	960 (197)	933.5 (186.5)
SARA (0–40)^b^	9.6 (2.8)	11.6 (4.5)	13.8 (5.2)	15.4 (6.3)
FARS total neuro (0–117)	42.6 (11.8)	48.7 (13.3)	53.9 (14.5)	56.8 (17.4)
Functional staging (0–6)	1.9 (1.0)	2.5 (1.1)	2.8 (1.0)	3.0 (1.2)
ADL (0–36)	5.4 (2.9)	8.0 (3.6)	9.9 (4.0)	11.7 (4.4)
9HPT dominant (s)	38.8 (9.8)	41.0 (11.2)	41.5 (10.7)	45.8 (12.2)
9HPT non-dominant (s)	43.1 (11.2)	47.8 (13.0)	50.2 (10.7)	55.3 (18.1)
Timed walk (s)^c^	5.8 (0.9)	5.8 (1.4)	6.0 (1.5)	6.4 (1.2)
Time from baseline (months)	0	13.7 (2.2)	26.8 (3.4)	45.2 (6.5)

Data are presented as mean (standard deviation).

a The shorter GAA length of an FRDA participant was not included due to a point mutation in one of the alleles.

b SARA score at baseline was not recorded for 15 of the 28 FRDA participants (including 10 out of 21 in the longitudinal FRDA cohort) due to SARA score being added later in the study.

c Two outliers were excluded: one participant had no value because they could not walk while another completed the walk in 105 s.

Individuals with FRDA were clinically evaluated with the original Friedreich Ataxia Rating Scale (FARS)^26^ and the Scale for the Assessment and Rating of Ataxia (SARA).^27^ SARA score was not collected for 15 individuals with FRDA at baseline.

### MRI protocol

The following imaging protocol was used at baseline for all recruited individuals (FRDA and controls) and subsequently repeated for individuals with FRDA during their follow-up visits. MRI measurements were performed on a 3 Tesla Siemens MAGNETOM Trio scanner (Siemens, Erlangen, Germany) with Syngo software version VD13D using the body transmit radio frequency coil and a 32-channel receive head coil array for brain imaging. During the study, the scanner was upgraded to a MAGNETOM Prisma^fit^ scanner with Syngo software version VE11C. The body transmit coil and a 64-channel receive head-neck coil were used after the upgrade. The potential impact of this upgrade was carefully investigated, and additional scans were performed before and after the upgrade to rule out potential bias on the results (see the Effect of scanner upgrade section). Results in the spinal cord have been reported in a separate paper.^20^

3D T_1_-weighted magnetization-prepared rapid gradient-echo (MPRAGE) images of the brain were acquired for morphometric analyses, with the following parameters: TR = 2530 ms, TE = 3.65 ms, TI = 1100 ms, flip angle = 7^°^, voxel size = 1 mm isotropic, FOV = 256 × 176 mm^2^, 224 coronal slices, GRAPPA = 2.

An echo-planar spin-echo sequence was used to acquire diffusion data in 128 directions with the following parameters: TR = 4253 ms, TE = 90.6 ms, voxel size = 1.8 mm isotropic, FOV = 192 × 192 mm^2^, 90 axial slices, multiband acceleration factor MB = 3, phase-encoding = Anterior–Posterior, *b*-value = 1500 s/mm^2^. Additionally, 17 interleaved non-diffusion-weighted images (*b*0) were acquired. The acquisition was repeated to improve the signal-to-noise ratio. Two additional datasets were also acquired with reversed phase-encoding (PA) to correct for geometric and eddy-current distortions. Total acquisition time for all four datasets was 42 min.

### Data analysis

#### Morphometry

One brain T_1_ dataset from the FRDA group at baseline was excluded due to significant motion artefacts. There were thus 27 brain T_1_ datasets at baseline, and 21, 19 and 14 datasets at the 1-year, 2-year and 3-year follow-up visits. The 3D T_1_-weighted images were processed with FreeSurfer version 6. The longitudinal data were processed with the longitudinal FreeSurfer pipeline that has been shown to reduce morphological variability within subjects for longitudinal data.^28^ A template was created for each subject using the data from all time points, and each time point data was then registered to this template to extract longitudinal volume measures. A Bayesian segmentation algorithm was applied to robustly segment the brainstem.^29^ Since FreeSurfer segmentation of the cerebellum is limited,^30^ an improved cerebellum segmentation algorithm, CERES,^31^ was instead used to segment the cerebellum. The morphometric measures were normalized by the total intracranial volume of each participant.

#### Microstructure

One baseline diffusion dataset and one 1-year follow-up diffusion dataset from the FRDA group were excluded due to significant artefacts. One more 1-year follow-up diffusion dataset from the FRDA group was excluded due to large motion artefacts that could not be optimally corrected. Therefore, there were 27 brain diffusion datasets at baseline, 19 at 1-year follow-up, 19 at 2-year follow-up and 14 at 3-year follow-up. Preprocessing of diffusion data included removing noise^32^ and Gibbs-ringing artefacts.^33^ Using FSL 6.01 (‘topup’ and ‘eddy’) with outliers replacement^34^ and slice-to-volume motion correction,^35^ the data were corrected for motion, susceptibility and eddy-current distortions.^36,37^ Furthermore, the B_1_ field inhomogeneity in the data was corrected with a nonuniform intensity normalization algorithm.^38^

##### Diffusion tensor imaging

The corrected data were then fitted to the diffusion tensor model, and DTI metrics (FA, fractional anisotropy; MD, mean diffusivity; RD, radial diffusivity; AD, axial diffusivity) were extracted.

##### Fixel-based analysis

This technique was used to overcome the limitations of the conventional DTI model, and to allow the differentiation of specific fibre populations in each voxel.^25^ The corrected data were first resized by a factor of 2 to increase the contrast-to-noise ratio. An unsupervised method that does not require segmentation of a T_1_ image was subsequently used to estimate the tissue-specific response function,^39^ counting the *b*0 image as a second shell to allow treating our single-shell data as a multi-shell data. To enhance the white matter (WM) signal and reduce WM volume overestimation, multi-tissue constrained spherical deconvolution (CSD)^40^ was applied using the average baseline response functions of WM and cerebrospinal fluid (CSF) (from all controls and patients for the cross-sectional analysis, and from patients only for the longitudinal analysis) to estimate the FODs. Intensity inhomogeneities in the FODs were further corrected with a multi-tissue informed log-domain intensity normalization to ensure a comparable absolute amplitude across all subjects.

Next, a study-specific unbiased population template was created from the normalized FODs of 15 controls and 15 patients for cross-sectional analysis. Another template was created from the normalized FODs of all patients for longitudinal analysis. The normalized FODs of individual participants, in each analysis category, were then registered to their respective templates. The brain mask of each individual subject was warped to the template space, and the intersection of all these masks was used to create the template mask in which subsequent analysis was performed. This ensured that the same regions were compared across subjects. The template FODs amplitudes within the template mask were calculated and thresholded at 0.06 to remove residual grey matter peaks, resulting in a corresponding image known as ‘template analysis fixel mask’. Individual FODs were then segmented to identify the number and orientation of fixels (i.e. individual fibre population) in each voxel and reoriented to the template space. Furthermore, the fixels of each individual were assigned to a corresponding fixel in the template space through a fixel–fixel correspondence that matches fixels across all subjects to a common set of template fixels in order to generate the fibre density (FD) maps. Using the deformation fields generated during the registration of FODs to the template space, the fibre cross-section (FC) is generated to encompass fibre bundles that cover multiple voxels. FD and FC were multiplied to obtain a combined measure of fibre density and cross-section (FDC). To guide the analysis of related fixels, a whole brain tractogram from the population template in each group was generated using 20 million tracts, and filtered^41^ to 2 million tracts to reduce biases and produce more anatomically meaningful white matter tracts.

#### Effect of scanner upgrade

Due to the scanner upgrade from Trio to Prisma, 15 control datasets were acquired on Trio and 5 on Prisma (baseline only). For participants with FRDA, 23 were scanned on Trio and 5 on Prisma at baseline. Of the 23 scanned on Trio at baseline, 18 were scanned at 1-year follow-up, with 11 on Trio and 7 on Prisma. Of the 5 scanned on Prisma at baseline, 3 were scanned at 1-year follow-up, all on Prisma. All 2-year and 3-year follow-up datasets were acquired on Prisma.

Five additional volunteers were scanned before and after the scanner upgrade to estimate the effect of the upgrade. Results show that most metrics remained stable after the upgrade (Supplementary Table 1). We note that this comparison has limited statistical power to detect very small differences. Previous studies have shown that upgrades can introduce a small but significant bias in longitudinal follow-up,^42-44^ and we have observed a similar trend in our data. For example, for the total cerebellar volume, nearly all ‘Trio-Prisma’ pairs showed an increasing trend while most ‘same scanner’ pairs of data points (Trio-Trio or Prisma-Prisma) showed a decreasing trend (Supplementary Fig. 1). The mean annual change in total cerebellar volume was −0.7% for ‘Same Scanner’ pairs and +1.3% for ‘Trio-Prisma’ pairs (unpaired two-sided *t*-test, *P* = 2 × 10^−9^). This small but systematic difference between Trio and Prisma was likely due to higher SNR in the lower part of the brain (including the cerebellum) with the 64-ch head and neck coil used on Prisma compared to the 32-ch head coil used on Trio. Therefore, for each individual subject, we only kept the data from the same scanner (either Trio data points or Prisma data points, whichever number of points was highest) for longitudinal analysis. For subjects who had two Trio and two Prisma data points, we kept all data, and the slope was calculated as the average of the slope for the two Trio points and the slope for the two Prisma points.

### Statistical analysis

#### Cross-sectional analysis

The FreeSurfer and CERES analyses yielded volume measurements of 25 and 15 regions of interests (ROIs), respectively (Table 2 and Supplementary Table 2). To extract DTI metrics, the John Hopkins University white matter atlas was registered to the native space of each individual using Advanced Normalization Tools (ANTs) (https://stnava.github.io/ANTs/). Mean values from 27 selected ROI were then extracted (Table 2 and Supplementary Table 3). A similar procedure was used to extract FBA metrics from the same 27 ROIs, and ANTs was used to register the atlas to the native space of each individual, which was then converted into a fixel mask to extract FBA metrics (Table 2 and Supplementary Table 4).

**Table 2:** Summary of cross-sectional results for selected variables

Metric	Control	FRDA	Difference (%)	Cohen’s *d*	*P*-value	
					Raw	Corrected
**Morphometry—FreeSurfer (10^−2^)**						
Medulla	0.322 ± 0.031	0.256 ± 0.018	−20.5%	−2.77	**4.02** × **10**^−^**^12^**	**1.01** × **10**^−^**^10^**
Pons	0.910 ± 0.080	0.830 ± 0.076	−8.8%	−1.03	**0.001**	**0**.**023**
Midbrain	0.409 ± 0.032	0.370 ± 0.017	−9.6%	−1.62	**1.87** × **10**^−^**^6^**	**4.31** × **10**^−^**^5^**
Thalamus	1.020 ± 0.064	0.960 ± 0.057	−5.9%	−1.01	**0.001**	**0**.**029**
Fourth ventricle	0.135 ± 0.028	0.159 ± 0.040	17.6%	0.67	**0.027**	0.483
SCP	0.015 ± 0.002	0.011 ± 0.001	−25.1%	−2.00	**2.36** × **10**^−^**^8^**	**5.66** × **10**^−^**^7^**
CSF	0.110 ± 0.012	0.123 ± 0.022	12.2%	0.73	**0.017**	0.335
VentralDC	0.532 ± 0.038	0.507 ± 0.033	−4.7%	−0.72	**0.019**	0.355
CC mid-posterior	0.029 ± 0.007	0.025 ± 0.005	−12.7%	−0.64	**0.036**	0.606
**Morphometry—CERES (10^−2^)**						
Cerebellum	8.278 ± 0.793	8.098 ± 0.563	−2.2%	−0.28	0.311	1.000
Cerebellum WM	0.938 ± 0.101	0.842 ± 0.084	−10.3%	−1.05	**3.0** × **10**^−^**^4^**	**0**.**005**
Cerebellum GM	7.339 ± 0.701	7.256 ± 0.512	−1.1%	−0.15	0.580	1.000
DTI—FA						
SCP	0.559 ± 0.045	0.437 ± 0.055	−21.8%	−2.38	**2.64** × **10**^−^**^10^**	**7.14** × **10**^−^**^9^**
ICP	0.390 ± 0.031	0.313 ± 0.038	−19.7%	−2.2	**2.02** × **10**^−^**^9^**	**5.24** × **10**^−^**^8^**
PLIC	0.623 ± 0.014	0.604 ± 0.022	−3.2%	−1.02	**0.001**	**0**.**024**
SCR	0.457 ± 0.016	0.437 ± 0.020	−4.4%	−1.07	**7.21** × **10**^−^**^4^**	**0**.**017**
Fx_ST	0.546 ± 0.028	0.517 ± 0.021	−5.4%	−1.21	**1.73** × **10**^−^**^4^**	**0**.**004**
**DTI—RD (10^−3^ mm^2^/s)**						
SCP	0.595 ± 0.064	0.789 ± 0.090	32.6%	2.42	**1.59** × **10**^−^**^10^**	**4.29** × **10**^−^**^9^**
ICP	0.821 ± 0.048	0.967 ± 0.121	17.7%	1.49	**7.12** × **10**^−^**^6^**	**1.85** × **10**^−^**^4^**
PLIC	0.370 ± 0.014	0.394 ± 0.026	6.5%	1.1	**5.05** × **10**^−^**^4^**	**0**.**012**
SCR	0.475 ± 0.022	0.496 ± 0.024	4.5%	0.91	**0.003**	0.073
Fx_ST	0.521 ± 0.038	0.561 ± 0.028	7.6%	1.21	**1.77** × **10**^−^**^4^**	**0**.**004**
**DTI—MD (10^−3^ mm^2^/s)**						
SCP	0.913 ± 0.052	1.053 ± 0.072	15.3%	2.17	**2.93** × **10**^−^**^9^**	**7.91** × **10**^−^**^8^**
ICP	1.020 ± 0.046	1.130 ± 0.115	10.7%	1.17	**2.32** × **10**^−^**^4^**	**0**.**006**
PLIC	0.636 ± 0.014	0.658 ± 0.022	3.4%	1.15	**3.10** × **10**^−^**^4^**	**0**.**008**
SCR	0.646 ± 0.024	0.663 ± 0.023	2.6%	0.73	**0.017**	0.347
Fx_ST	0.790 ± 0.033	0.823 ± 0.030	4.2%	1.06	**8.05** × **10**^−^**^4^**	**0**.**019**
**DTI—AD (10^−2^ mm^2^/s)**						
SCP	0.155 ± 0.006	0.158 ± 0.007	2.1%	0.51	0.090	1
ICP	0.142 ± 0.006	0.146 ± 0.011	2.7%	0.41	0.172	1
PLIC	0.117 ± 0.002	0.119 ± 0.003	1.4%	0.64	**0.034**	0.891
SCR	0.099 ± 0.004	0.100 ± 0.003	0.9%	0.27	0.372	1
Fx_ST	0.133 ± 0.004	0.135 ± 0.005	1.6%	0.45	0.137	1
**FBA—FD**						
SCP	0.889 ± 0.054	0.711 ± 0.066	−20.0%	−2.89	**9.16** × **10**^−^**^13^**	**7.23E-11**
ICP	0.743 ± 0.045	0.677 ± 0.049	−9.0%	−1.41	**1.96** × **10**^−^**^5^**	**0**.**001**
PLIC	1.030 ± 0.030	0.994 ± 0.034	−3.4%	−1.1	**5.45** × **10**^−^**^4^**	**0**.**035**
SCR	0.833 ± 0.031	0.780 ± 0.030	−6.3%	−1.73	**4.89** × **10**^−^**^7^**	**3.57** × **10**^−^**^5^**
**FBA—FC**						
SCP	0.045 ± 0.058	−0.171 ± 0.083	-	−2.93	**5.92** × **10**^−^**^13^**	**4.74** × **10**^−^**^11^**
ICP	0.063 ± 0.062	−0.112 ± 0.070	-	−2.61	**1.92** × **10**^−^**^11^**	**1.48** × **10**^−^**^9^**
PLIC	0.014 ± 0.061	−0.081 ± 0.066	-	−1.48	**8.45** × **10**^−^**^6^**	**6.00** × **10**^−^**^4^**
SCR	0.024 ± 0.087	−0.089 ± 0.078	-	−1.38	**2.58** × **10**^−^**^5^**	**0**.**002**
**FBA—FDC**						
SCP	0.937 ± 0.093	0.603 ± 0.092	−35.7%	−3.61	**6.54** × **10**^−^**^16^**	**5.30** × **10**^−^**^14^**
ICP	0.797 ± 0.077	0.608 ± 0.065	−23.7%	−2.7	**8.45** × **10**^−^**^12^**	**6.59** × **10**^−^**^10^**
PLIC	1.056 ± 0.091	0.920 ± 0.068	−12.9%	−1.73	**5.04** × **10**^−^**^7^**	**3.63** × **10**^−^**^5^**
SCR	0.867 ± 0.097	0.718 ± 0.061	−17.2%	−1.91	**6.41** × **10**^−^**^8^**	**4.81** × **10**^−^**^6^**

Data are presented as mean ± standard deviation. Volumes are normalized by total intracranial volume. Bold values are significant *P*-values.

ROI-based measures were fitted with linear models with age and sex as covariates to compare results between the control and FRDA groups. Correction for multiple comparison was performed across regions within each parameter (i.e. volumes from FreeSurfer and CERES, and diffusion metrics FA, MD, AD, RD, FC, FD and FDC) using step-down Holm–Bonferroni.

Tract-based spatial statistics (TBSS)^45^ with 5000 permutations were also used to compare DTI metrics between the control and FRDA groups using family-wise error correction. However, due to the less optimized default FA-based spatial normalization of TBSS, we performed a tensor-based spatial normalization of the DTI maps using DTI-TK^46^ before running voxel-wise statistics. We observed that the use of tensor-based registration improved the alignment of dMRI data compared to the standard scalar-based registration (Supplementary Fig. 2) for extraction of accurate metrics.

Connectivity-based fixel enhancement,^47^ a threshold-free cluster enhancement approach, was used to perform voxel-wise statistical comparison of FBA metrics between the control and FRDA groups.

#### Longitudinal analysis

To reduce the number of multiple comparisons for longitudinal analysis, brain regions were selected a priori based on high cross-sectional effect size (Cohen’s *d*) at baseline. In doing so, we hypothesized that the largest longitudinal effect size would be in the regions that were most affected in participants with FRDA patients versus controls (see Discussion). Specifically, the top nine ROIs with absolute Cohen’s *d* > 0.6 were selected for FreeSurfer morphometry: medulla, pons, midbrain, thalamus, fourth ventricle, SCP, CSF, ventralDC and CC mid-posterior. For CERES, we focused on the cerebellar, cerebellar WM and cerebellar GM ROIs because differences have been reported for these three ROIs at later disease stage. Similarly, five DTI ROIs were selected, with consistently high Cohen’s *d* (>0.9) for both FA and RD (i.e. SCP, ICP, PLIC, SCR and Fx_ST). Cross-sectional differences in AD were much smaller, and as a result, both AD and MD generally had smaller cross-sectional effect size than RD for any given region. Therefore, we did not include AD and MD in a priori variables for longitudinal analysis. Four of those ROIs (SCP, ICP, PLIC and SCR) also showed consistently high Cohen’s *d* (>0.9) for all three FBA metrics FD, FC and FDC and were therefore selected for longitudinal analyses.

To extract the longitudinal DTI metrics (FA and RD from five ROIs), the same procedure for registration to the John Hopkins University white matter atlas was used as reported for the cross-sectional analysis. ANTs was also used to register the John Hopkins University atlas to the longitudinal FBA template space and then converted into a fixel mask to extract longitudinal FBA metrics (FD, FC and FDC) in the selected four ROIs.

Longitudinal slopes (change over time) were estimated for each MR metric and each subject with linear regression using ‘same scanner’ time points for each subject as described above. The mean slope for each MR metric was calculated by averaging slopes across subjects. One-sided one-sample *t*-tests were used to statistically evaluate the slopes, hypothesizing that the changes over time would go in the same direction as the cross-sectional differences. Correction for multiple comparison was performed across regions within each parameter using step-down Holm–Bonferroni. The sample size of the longitudinal cohort (*n* = 21) was sufficient to detect slopes >0 (or <0) with an effect size of 0.5 (or −0.5) or better, with α = 0.05 and β = 0.7.

In graphs showing the longitudinal data, the Prisma values were adjusted by a correction factor to eliminate the bias due to the scanner upgrade. This correction factor (different for each metric) was calculated as the difference between the mean 1-year change for all ‘same scanner’ pairs of data points (‘Trio-Trio’ or ‘Prisma-Prisma’) and the mean 1-year change for all ‘Trio-Prisma’ pairs of data points. For example, for total cerebellum volume, the mean 1-year change for all ‘same scanner’ pairs was −0.7%, and the mean 1-year change for all ‘Trio-Prisma’ pairs was +1.3% (Supplementary Fig. 1). Therefore, the Prisma values were adjusted by −2%. Note that this correction factor was applied only for display purposes. The longitudinal slopes were calculated from same scanner data only and without correcting Prisma values.

#### Associations between MRI and clinical metrics

We used linear mixed models to test for associations between MR metrics and clinical metrics in patients, using patient data only and including data from all time points, with subjects as random variable to account for repeated measures (multiple time points for each subject). This analysis was performed for the same MR variables selected for longitudinal analysis. Significance testing for these associations is presented along with correlations for ease of interpretation of magnitudes and directions of effects.

## Results

The most significant cross-sectional results (corresponding to variables subsequently selected a priori for the longitudinal analysis) are summarized in Table 2, and the complete cross-sectional results are provided in Supplementary Tables 2–4. Longitudinal results are summarized in Table 3, and the complete longitudinal results are provided in Supplementary Table 5. Associations between MR metrics and clinical metrics are provided in Table 4. Bold values in Tables 2–4 represent *P* ≤ 0.05.

**Table 3:** Longitudinal slope showing the annual change (over 3 years) in imaging and clinical parameters

Metric	Mean	SD	Δ from baseline	SRM	*P*-value	
					Raw	Corrected
**Morphometry—FreeSurfer**						
Medulla	−0.000013	0.000047	−0.5%	−0.28	0.109	0.218
Pons	−0.000053	0.000087	−0.7%	−0.60	**0**.**006**	**0**.**035**
Midbrain	−0.000025	0.000041	−0.7%	−0.60	**0**.**006**	**0**.**030**
Thalamus	−0.000175	0.000211	−1.9%	−0.83	**5.61 × 10** ^−^ **^4^**	**0**.**004**
Fourth ventricle	0.000065	0.000076	3.9%	0.86	**4.21 × 10^−4^**	**0**.**004**
SCP	−0.000002	0.000006	−2.1%	−0.40	**0**.**041**	0.165
CSF	0.000017	0.000057	1.3%	0.30	0.091	0.272
VentralDC	−0.000046	0.000055	−0.9%	−0.85	**4.65 × 10^−4^**	**0**.**004**
CC mid-posterior	0.000003	0.000019	1.4%	0.17	0.218	0.218
**Morphometry—CERES**						
Cerebellum WM	−0.000083	0.000074	−1.0%	−1.12	**2.53 × 10^−5^**	**7.58 × 10^−5^**
Cerebellum	−0.000572	0.000517	−0.7%	−1.11	**2.87 × 10^−5^**	**5.73 × 10^−5^**
Cerebellum GM	−0.000490	0.000481	−0.7%	−1.02	**7.43 × 10^−5^**	**7.43 × 10^−5^**
**DTI—FA**						
SCP	−0.007770	0.013606	−1.7%	−0.57	**0**.**010**	**0**.**049**
ICP	−0.000680	0.019265	−0.2%	−0.04	0.438	0.876
PLIC	−0.000784	0.003736	−0.1%	−0.21	0.180	0.720
SCR	0.000049	0.004100	0.0%	0.01	0.479	0.479
Fx_ST	−0.001470	0.008479	−0.3%	−0.17	0.224	0.671
**DTI—RD**						
SCP	0.000021	0.000022	2.7%	0.97	**1.83 × 10^−4^**	**9.13 × 10^−5^**
ICP	0.000045	0.000074	4.7%	0.61	**0**.**007**	**0**.**028**
PLIC	−0.000001	0.000004	−0.2%	−0.19	0.202	0.606
SCR	−0.000001	0.000004	−0.1%	−0.13	0.284	0.567
Fx_ST	0.000001	0.000011	0.2%	0.07	0.373	0.373
**FBA—FD**						
SCP	−0.014312	0.025843	−1.9%	−0.55	**0**.**011**	**0**.**023**
ICP	0.005337	0.041857	0.8%	0.13	0.288	0.288
PLIC	−0.006910	0.011755	−0.7%	−0.59	**0**.**008**	**0**.**025**
SCR	−0.006384	0.007644	−0.8%	−0.84	**0**.**001**	**0**.**003**
**FBA—FC**						
SCP	−0.017919	0.021921		−0.82	**0**.**001**	**0**.**002**
ICP	−0.009043	0.018887		−0.48	**0**.**023**	**0**.**023**
PLIC	−0.015104	0.012951		−1.17	**2.46 × 10^−5^**	**9.84 × 10^−5^**
SCR	−0.019465	0.021621		−0.90	**3.61 × 10^−4^**	**0**.**001**
**FBA—FDC**						
SCP	−0.026530	0.025556	−3.7%	−1.04	**8.88 × 10^−5^**	**3.55 × 10^−4^**
ICP	−0.000328	0.035886	−0.1%	−0.01	0.484	0.484
PLIC	−0.021052	0.021666	−2.3%	−0.97	**1.21 × 10^−4^**	**3.62 × 10^−4^**
SCR	−0.021441	0.021275	−2.9%	−1.01	**1.74 × 10^−4^**	**3.48 × 10^−4^**
**Clinical**						
ADL	1.954783	1.324931	26%	1.48	**7.07 × 10^−7^**	**3.53 × 10^−6^**
FARS total neuro	4.930495	4.244949	10%	1.16	**1.64 × 10^−5^**	**6.58 × 10^−5^**
SARA	2.068959	1.809986	17%	1.14	**1.17 × 10^−4^**	**2.35 × 10^−4^**
Functional staging	0.330311	0.328952	14%	1.00	**8.64 × 10^−5^**	**2.59 × 10^−4^**
9HPT non-dominant	2.953038	4.731900	6%	0.62	**0**.**005**	**0**.**005**

Volumes are normalized by total intracranial volume. Bold values are significant *P*-values.

**Table 4:** Correlation of imaging metrics with clinical scores

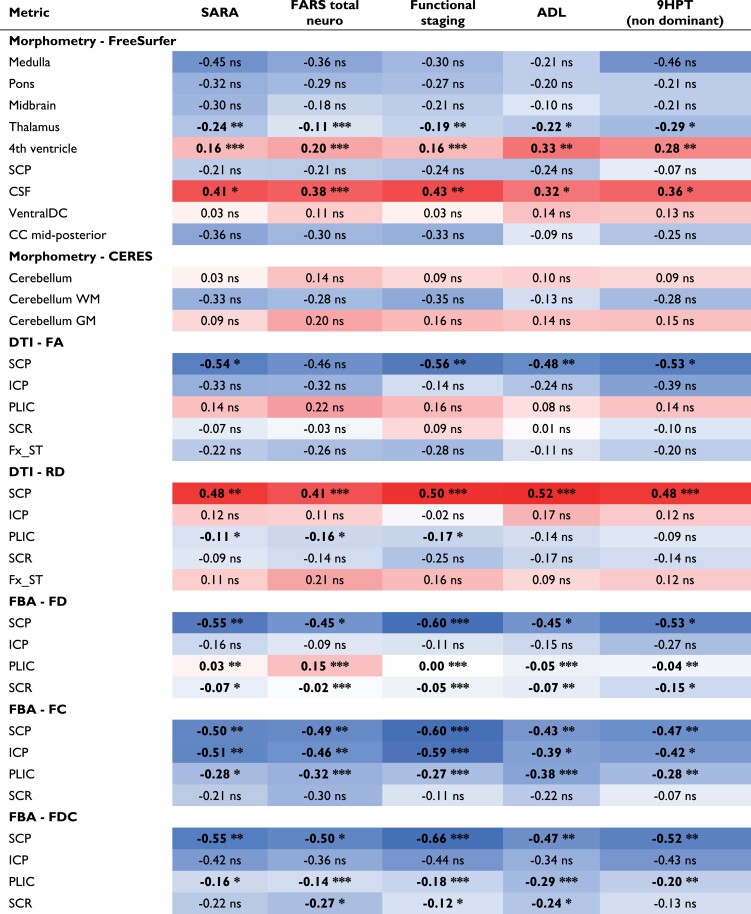

Data are the *R*-values corresponding to Pearson correlation coefficients using all time points; *P*-values shown correspond to linear models of the same associations that account for the repeated measurements within person. Bold values represent statistical significance. Blue, negative correlation; red, positive correlation.

*P < 0.05.

**P < 0.01.

***P < 0.001.

### Morphometry

**Figure 1:**
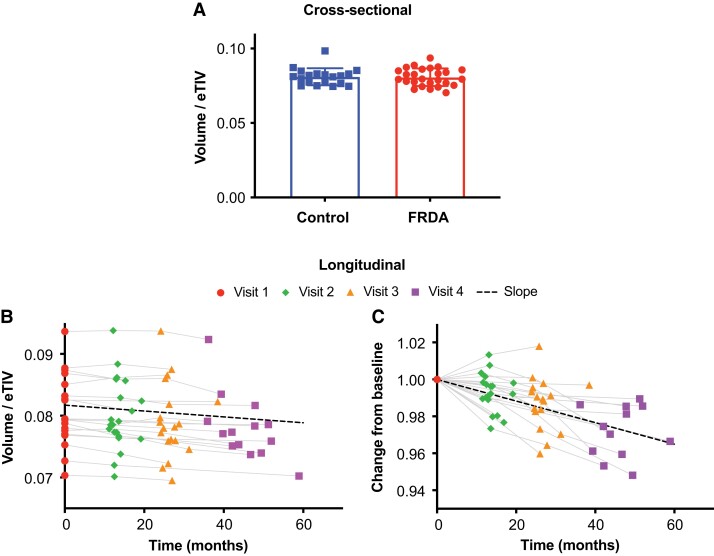
**Cross-sectional differences and longitudinal changes in the cerebellar volume of FRDA participants. A)** There was no significant difference in total cerebellum volume in FRDA participants compared to controls at baseline (linear model *t*-test of FRDA versus control coefficient, *P* = 1). **B)** Total cerebellum volume showed a significant decrease over time in patients (one-sided one-sample *t*-test on slopes, *P* = 5.73 × 10^−5^). **C)** Same data as **B**, but visualized differently with each subject normalized to 1 at baseline. The change corresponded to a −0.7% annual decrease in total cerebellum volume. eTIV, estimated total intracranial volume.

Compared to controls, individuals with FRDA showed reduced volumes in the medulla (−20.5%), superior cerebellar peduncles (SCPs, −25.1%), midbrain (−9.6%), pons (−8.8%), thalamus (−5.9%) and ventral diencephalon (−4.7%). Also, from the robust CERES method, we observed reduced volume in the cerebellum WM (−10.3%) and lobule IV (−7.7%). Additionally, we found increased volumes of the fourth ventricle (17.6%) and total cerebrospinal fluid (CSF, 12.2%). After correction for multiple comparisons, the medulla (Cohen’s *d* = −2.77, *P* = 1.01 × 10^−10^), SCP (Cohen’s *d* = −2.00, *P* = 5.66 ×10^−7^), midbrain (Cohen’s *d* = −1.62, *P* = 4.31 × 10^−5^), pons (Cohen’s *d* = −1.03, *P* = 0.023), thalamus (Cohen’s *d* = −1.01, *P* = 0.029) and cerebellar white matter (Cohen’s *d* = 1.05, *P* = 0.005) remained significant (Table 2 and Fig. 1).

Longitudinally, the largest effect size was observed for the total cerebellar white matter with a −1.0% annual volume change (*P* = 7.58 × 10^−5^) and a standardized response mean (SRM) of −1.12 using CERES (Table 3). Interestingly, significant annual change was also found for the total cerebellum (−0.7%/year, *P* = 5.73 × 10^−5^, SRM = −1.11) (Table 3 and Fig. 1) and total cerebellar grey matter (−0.7%/year, *P* = 7.43 × 10^−5^, SRM = −1.02), even though those two variables showed almost no atrophy compared to controls at baseline (−2.2% and −1.1% lower volume, respectively, n.s.) (Table 3). Similar results were obtained with FreeSurfer, but with smaller effect size, confirming that CERES is more precise than FreeSurfer for cerebellum morphometry. The thalamus and ventral diencephalon also showed significant annual volume change of −1.9% (SRM = −0.83, *P* = 0.004) and −0.9% (SRM = −0.85, *P* = 0.004), respectively. The pons and midbrain had lower SRM (−0.6 for both, *P* = 0.035 and *P* = 0.030, respectively) and annual volume change of −0.7%. The fourth ventricle had the largest annual volume change (+3.9%) with a relatively high SRM of 0.86 (*P* = 0.004). Longitudinal volume change was not statistically significant in the medulla (−0.5%, *P* = 0.218), SCP (−2.1%, *P* = 0.165) or in total CSF (+1.3%, *P* = 0.272).

### Microstructure

#### Diffusion tensor imaging

**Figure 2:**
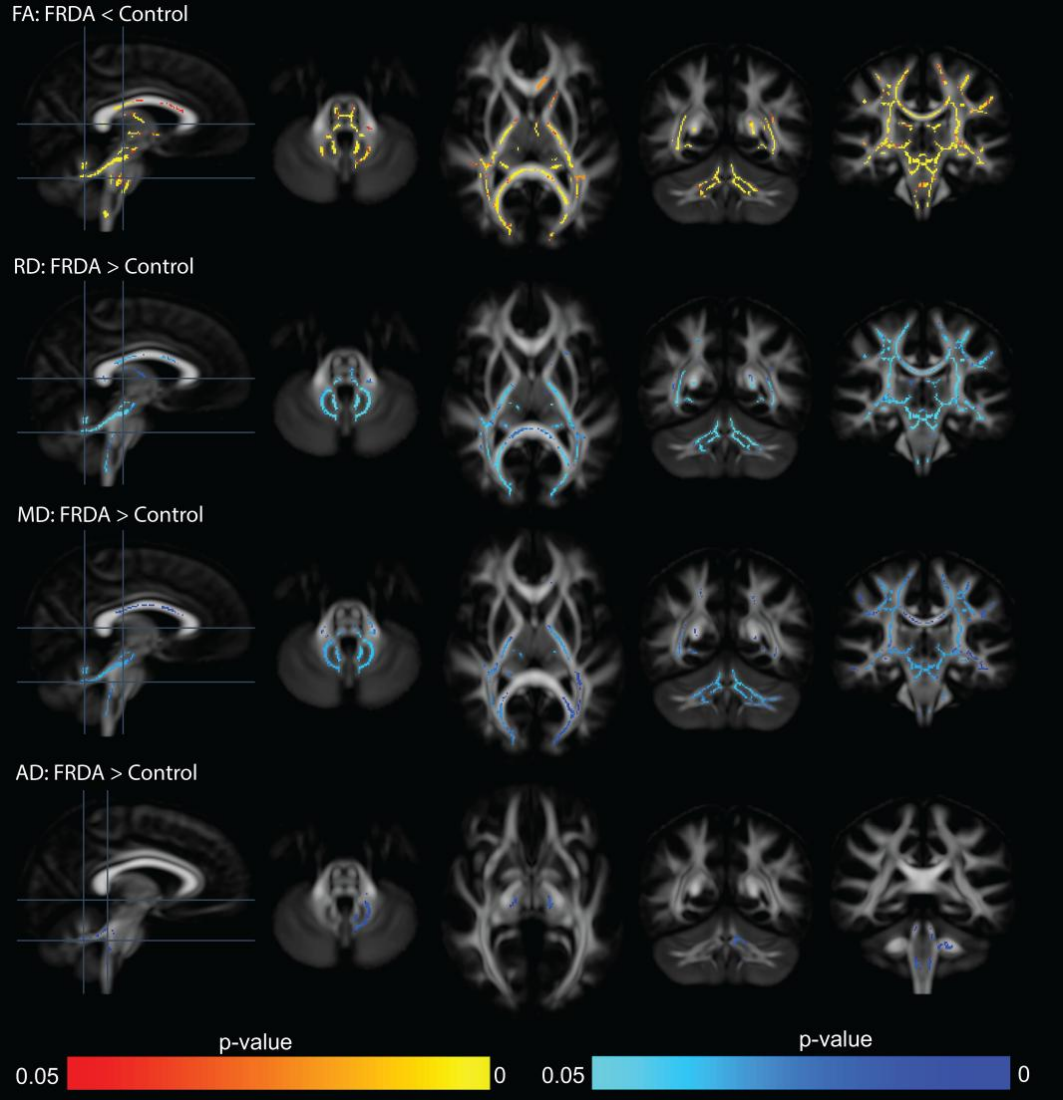
**Cross-sectional tract-based spatial statistics for diffusion tensor imaging metrics reveal altered brain white matter microstructure in individuals with FRDA compared to controls.** Lower fractional anisotropy (FA) and higher mean diffusivity (RD), radial diffusivity (MD) and axial diffusivity (AD) were observed in several brain regions. While AD differences were limited to the cerebellar peduncles, differences in FA, RD and MD were pronounced in both the cerebellar and cerebral white matter.

Compared to controls, TBSS revealed that individuals with FRDA had lower FA and higher RD and MD in many white matter areas, including the corpus callosum, thalamic radiations, internal capsule, corona radiata, longitudinal fasciculus, sagittal stratum, corticospinal tract, medial lemniscus, pontine crossing tract, fornix/stria terminalis, cerebral peduncles and cerebellar peduncles (Fig. 2). Differences in AD were much more limited, with higher AD in the FRDA group only in the cerebellar peduncles (Fig. 2).

**Figure 3:**
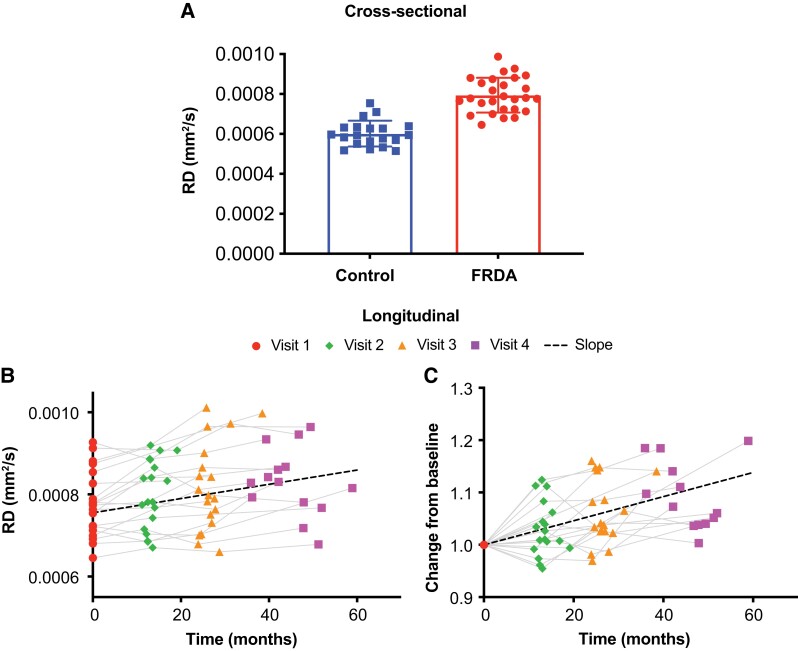
**Cross-sectional differences and longitudinal changes in radial diffusivity in the superior cerebellar peduncles of FRDA participants. A)** Compared to controls, FRDA participants exhibited significantly higher radial diffusivity (RD) in the superior cerebellar peduncles (linear model *t*-test of FRDA versus control coefficient, *P* = 4.29 × 10^−9^). **B)** Radial diffusivity showed a significant increase over time in patients (one-sided one-sample *t*-test on slopes, *P* = 9.13 × 10^−5^). **C)** Same data as **B**, but visualized differently with each subject normalized to 1 at baseline. The change corresponded to a 2.7% annual increase in radial diffusivity.

ROI-based analysis showed results that were in general consistent with the TBSS analysis, with the largest cross-sectional differences in the superior cerebellar peduncles (FA: −21.8%; RD: 32.6%) with high effect sizes (Cohen’s *d* =−2.38 and 2.42 for FA and RD, respectively), followed by the inferior cerebellar peduncles (FA: −19.7%; RD: 17.7%) also with relatively high effect sizes (Cohen’s *d* = −2.2 and 1.49 for FA and RD, respectively) (Table 2 and Fig. 3).

In FRDA participants, longitudinal TBSS analysis showed sparsely reduced FA and MD at 1-year follow-up, and reduced AD at 1-year follow-up in several regions (Supplementary Fig. 3). Due to the scanner upgrade, few subjects had baseline and 2-year or 3-year follow-up data acquired on the same scanner, therefore, longitudinal TBSS analysis was possible only for 1-year follow-up, thereby limiting the statistical power. Hence, our focus for longitudinal microstructural alterations was on the ROI-based analysis, which allowed more flexible statistical modelling. Longitudinal ROI-based analysis in the FRDA group showed a 1.7% annual decrease (SRM = −0.57, *P* = 0.049) in FA and a 2.7% annual increase in RD (SRM = 0.97, *P* = 9.13 × 10^−5^) in the SCP (Fig. 3). In the inferior cerebellar peduncles (ICP), there was a 4.7% annual increase in RD (SRM = 0.61, *P* = 0.028), but no significant change in FA (Table 3). The longitudinal ‘decrease’ in AD in many large white matter tracts with TBSS was unexpected, and was also observed in some ROI-based metrics not selected a priori for longitudinal analysis (see list of slopes for all variables in Supplementary Table 5). Note that some of longitudinal changes observed in ROI-based analyses (e.g. SCP) were not observed in the 1-year TBSS, consistent with our expectation that ROI-based analyses are more sensitive than voxel-based analyses.

#### Fixel-based analysis

FBA allows the extraction of different parameters that more directly reflect biological features such as fibre density and fibre cross-section, compared to DTI metrics.

**Figure 4:**
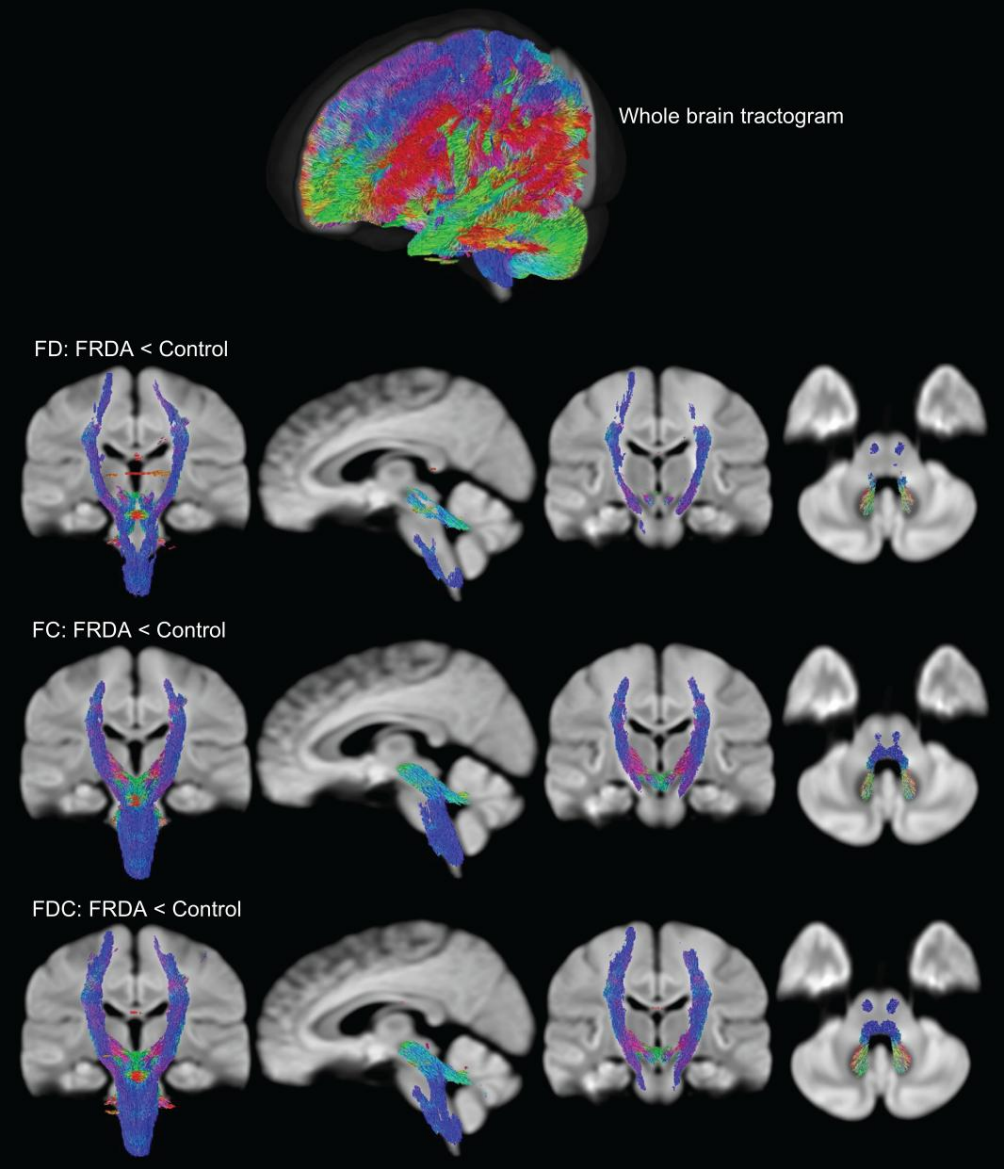
**Cross-sectional fixel-based analysis reveals altered fibre density (FD), fibre bundle cross-section (FC) and fibre density and cross-section (FDC) in individuals with FRDA compared to controls.** Lower values for these robust metrics were observed in similar brain regions in all three metrics. The streamlines have been directionally colour-coded (red, left–right; green, anterior–posterior; blue, superior–inferior).

**Figure 5:**
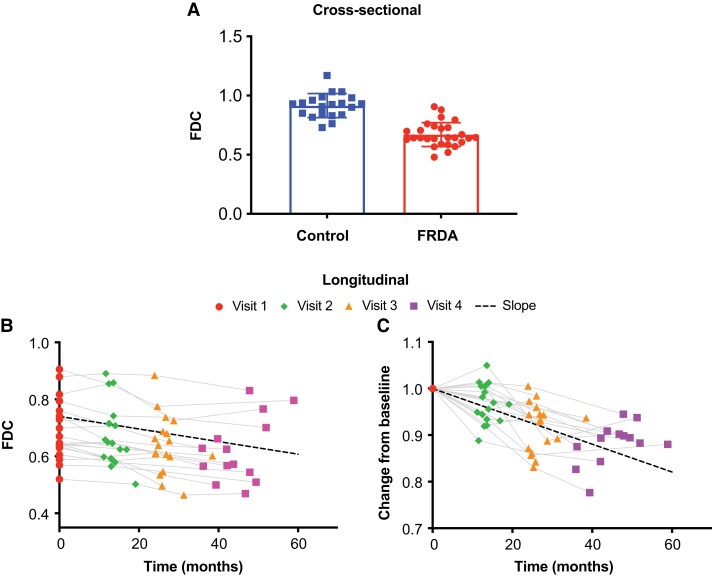
**Cross-sectional differences and longitudinal changes in fibre density and cross-section (FDC) in the superior cerebellar peduncles of FRDA participants. A)** Axonal alterations resulted in significantly lower FDC in FRDA participants compared to controls (linear model *t*-test of FRDA versus control coefficient, *P* = 5.30 × 10^−14^). **B)** FDC decreased significantly over time in patients (one-sided one-sample *t*-test on slopes, *P* = 3.53 × 10^−6^). **C)** Same data as **B**, but visualized differently with each subject normalized to 1 at baseline. The change corresponded to a −3.7% annual decrease in FDC.

Compared to controls, voxel-wise whole brain FBA analysis revealed that FD, FC and FDC of individuals with FRDA were significantly lower in the ICP, SCP, PLIC and SCR, as well as the corticospinal tract, medial lemniscus and cerebral peduncles (Fig. 4). ROI-based analysis (Table 2 and Fig. 5) revealed that the largest effect size for FD, FC and FDC was observed for the SCP (Cohen’s *d* = 2.89, 2.93 and 3.61, respectively) and ICP (Cohen’s *d* = 1.41, 2.61 and 2.7, respectively). In general, FDC presented larger effect sizes compared to FD and FC. The FBA findings are also largely consistent with those from DTI metrics, with even larger differences between groups and higher effect sizes.

Like the longitudinal TBSS analysis, longitudinal voxel-wise FBA analysis was also limited to baseline and 1-year follow-up datasets in FRDA participants to due to the scanner upgrade. There was reduced FD, FC and FDC in the corpus callosum, cerebral peduncle, internal capsule and corona radiata (Supplementary Fig. 4). FD was also decreased in the posterior thalamic radiation.

For longitudinal ROI-based analysis, FBA metrics appear to be more sensitive to changes than DTI metrics, and with higher effect sizes. For example, FDC was significantly reduced in PLIC (−2.3%/year, *P* = 3.62 × 10^−4^) and SCR (−2.9%/year, *P* = 3.48 × 10^−4^) with SRM = −0.97 and −1.01, respectively, but no significant longitudinal change was found in DTI metrics (FA and RD) in those regions (Table 3). For SCP, RD and FDC had similar SRM (−0.97 and −1.04, respectively) but there was greater annual change in FDC (−3.7%, *P* = 3.55 × 10^−4^) compared to RD. Overall, FDC appeared to be most sensitive to longitudinal change (around −3% per year depending on the white matter tract of interest) and with SRM around −1, approaching the longitudinal effect size of clinical scales SARA (SRM = 1.16) and FARS (SRM = 1.14). Note that some of longitudinal changes observed in ROI-based analyses (e.g. SCP) were not observed in the 1-year voxel-based FBA analysis, consistent with our expectation that ROI-based analyses are more sensitive than voxel-based analyses.

### Correlations between MRI and clinical metrics

Correlations between imaging and clinical metrics are summarized in Table 4.

Surprisingly, none of the cerebellar morphometry metrics were significantly correlated with any of the clinical metrics. However, CSF and fourth ventricle volumes were strongly positively correlated with all clinical metrics. The thalamus volume was also strongly negatively correlated with all clinical metrics, specifically the FARS total neuro and functional staging scores, and SARA.

Among the DTI metrics, FA and RD in the SCP strongly correlated (negatively and positively, respectively) with all clinical scales (except FARS total neuro score for FA). Interestingly, although no significant longitudinal change in RD was found in the posterior limb of the internal capsule, significant (albeit relatively weak) negative correlations with SARA, FARS total neuro and FARS functional staging score were found in this white matter tract.

Strong correlations were also identified between all FBA metrics and clinical scales. In particular, FD, FC and FDC of the SCP were consistently negatively correlated with all clinical scales. Similar findings were seen for FC and FDC of the posterior limb of the internal capsule, as well as for FC of the ICP.

We also note that no associations were found between GAA repeat numbers and MRI metrics.

## Discussion

This study reports cross-sectional and longitudinal macrostructural and microstructural alterations in the brain of individuals with early-stage FRDA. This is the first longitudinal study in an early-stage FRDA cohort, at a time when clinical progression (as measured by clinical scales such as SARA and FARS) can be rapid. In addition, this is the first study using FBA to evaluate individual fibre populations in FRDA. With this method, we report alterations in fibre density and cross-section that have not been previously identified.

A number of macrostructural and microstructural metrics displayed longitudinal sensitivity to disease progression and/or strong correlations with clinical metrics, making them potential candidate biomarkers for clinical trials. Many of the affected regions are part of the ascending spino-cerebellar tract or cerebello-thalamo-cortical tract.

Brain atrophy, as observed with cross-sectional brain morphometry, was most pronounced in the brainstem and cerebellar white matter (which includes the dentate nucleus) in individuals with FRDA. The brainstem connects the spinal cord with the cerebrum, and thus its pronounced atrophy (and continued degeneration as detected here for pons and midbrain) is consistent with the well-known pathology of the spinal cord in FRDA and with our recent findings indicating lower volume and altered microstructure in the cervical spinal cord in this same cohort.^20^ In contrast, total cerebellum volume and total cerebellar grey matter volume were not significantly different from healthy controls, as previously reported.^9,17^ Other studies have reported cerebellar grey matter differences at later stage.^6,9,12^ Our results confirm that there is very little atrophy in the cerebellum (except for cerebellar white matter) at early stage^12^ when compared to controls. The significant atrophy in cerebellar white matter is consistent with atrophy of the dentate nucleus.^48^ In the cerebrum, differences in deep grey matter (e.g. thalamus) volumes were observed, in line with previous reports.^8,11,14,23^

Several regions with significant atrophy in individuals with FRDA at baseline also showed significant longitudinal volume change. Factors such as larger cohort size or earlier disease stage could explain why such changes were not observed in previous studies.^8,21^ However, some metrics such as total CSF, corpus callosum and especially, medulla volumes, did not show significant longitudinal changes in FRDA in spite of large cross-sectional differences with controls. The lack of longitudinal change in medulla volume is particularly noteworthy and consistent with the developmental hypothesis of Friedreich,^49^ and more recent neuroimaging findings.^22^

The most prominent longitudinal change, so far largely overlooked in FRDA, was a ∼4% annual increase (SRM = 0.86) in the volume of the fourth ventricle, which lies dorsal to the brainstem and ventral to the cerebellum. Total cerebellum volume and total cerebellar grey matter volume also showed significant longitudinal change, even though they were not significantly different in FRDA, compared to controls, at baseline.^50^ In fact, total cerebellum volume had one of the highest longitudinal effect sizes (SRM = −1.11) among all reported morphometric results and showed a 0.7% annual decrease. The lack of significant cerebellar grey matter atrophy at baseline suggests that degeneration in cerebellar grey matter may begin at approximately the same time as the onset of symptoms and may be one of the underlying biological events associated with disease onset.

The SCP is another region that is particularly relevant in FRDA (and other inherited ataxias) as it is the largest cerebellar efferent bundle.^51^ Atrophy of the SCP in FRDA relative to controls has been reported previously.^11,52^ Longitudinally, we observed a ∼2% annual decrease in SCP volume but this did not reach significance after correction for multiple comparison. This could be due in part to the fact that FreeSurfer may not be optimized to accurately measure the small SCP volume. Other methods have been proposed to segment the SCP^11,52^ and may be more precise and result in less inter-subject variability. Among morphometric results that showed significant longitudinal changes, only thalamus, CSF and fourth ventricle volumes showed correlations with all clinical metrics.

Our modified TBSS analysis for DTI metrics revealed widespread cross-sectional white matter microstructural alterations in several cerebral and cerebellar regions. Our study took advantage of a tensor-based registration method with improved tensor alignment^46^ that can provide more robust results than the default scalar-based registration. We observed lower FA in several regions, which was associated with higher MD, RD and/or AD, similar to previous studies.^8,10,14,19^ These findings suggest neuronal damage and possible demyelination in these regions. However, these changes cannot be attributed to a specific biological mechanism due to limitations in the interpretation of DTI metrics,^24^ mostly due to the presence of crossing-fibres.^53^ To overcome this limitation, one study^23^ used tractography to detect FA alterations in specific pathway connecting the brainstem, dentate nucleus and cerebellar peduncles. Another study^17^ used CSD to resolve crossing-fibres in the dentatorubral and dentatothalamic tracts in order to report alterations in DTI metrics. However, these studies^17,23^ did not report on tract-specific measures that could lead to a more detailed biological interpretation, but rather used tractography to better delineate specific white matter regions from which to report DTI metrics. Here, we used FBA^25^ to resolve crossing-fibres and extract quantifiable tract-specific metrics with more meaningful biological interpretation and improved effect sizes (compared to DTI). Our results showed for the first time in FRDA that the intra-axonal volume fraction, also known as fibre density (FD), was significantly reduced for many white matter tracts (e.g. internal capsule, corona radiata, longitudinal fasciculus, corticospinal tract, medial lemniscus and cerebellar and cerebral peduncles) in individuals with FRDA. Furthermore, the fibre bundle cross-section (FC) that describes the fibre bundle morphology was also significantly reduced in individuals with FRDA. The combined measure (FDC) of intra-axonal volume fraction (FD) and fibre bundle morphology (FC) revealed significantly altered white matter microstructure in individuals with FRDA. Many of these affected structures are part of the ascending cerebello-thalamo-cortical tract. These findings show that widespread microstructural damage is already present at early stage.

Using an ROI-based analysis, longitudinal changes in DTI metrics were observed in the SCP (FA and RD) and ICP (RD). There was a nearly 2% annual decrease in FA and 3% annual increase in RD in the SCP, and an annual increase in RD in the ICP close to 5%. FA and RD in the SCP correlated with clinical scores, emphasizing the importance of the SCP in FRDA. FBA metrics demonstrated higher sensitivity and effect sizes. They identified significant longitudinal reductions in the SCP, posterior limb of the internal capsule and superior corona radiata. While annual changes in SCP diffusion metrics had similar absolute SRM (around 1.0) for DTI (RD) and FBA (FDC), annual changes in PLIC and SCR were much more robust for FBA. Furthermore, FC showed longitudinal decrease in the ICP. These longitudinal metrics also showed strong correlations with all clinical scores. These findings support the sensitivity of these microstructural metrics and their potential to follow disease progression.

Together, our findings in the brain in the present paper and our previously published findings in the spinal cord in the same cohort^20^ provide a comprehensive picture of the nervous system in FRDA at an early stage of the disease: cross-sectionally, morphometric data show large alterations in FRDA compared to controls in the spinal cord (∼−30% in cross-sectional area), the medulla (−20%), in the SCP (−25%), and to a lesser extent the mid-posterior corpus callosum (−12.7%), midbrain (−9.6%), pons (−8.8%), the cerebellar WM (−5.9%) and the thalamus (−5.9%). These structures are part of the ascending spino-cerebello-thalamo-cortical pathway: the medulla connects to the cerebellum via the ICP and gives rise to the spinal cord, while the SCP connects to the cerebellar hemispheres to the thalamus. The rest of the brain shows few changes, consistent with the fact that the brain appears mostly normal in FRDA and that some of these changes likely appear well before disease onset due to abnormal development (hypoplasia). Microstructural metrics of the brain and spinal cord show a similar pattern, with the largest cross-sectional changes in diffusion metrics observed in the spinal cord, SCP and ICP.

Longitudinally, we found the largest morphometric effect size (SRM) for the cerebellar volumes (total, WM and GM), even though there was little atrophy at baseline for the total cerebellum and cerebellar GM, suggesting that neurodegeneration of the cerebellar grey matter may play an important role in the development of symptoms. For microstructure, the largest effect sizes were observed in SCP (RD and FDC), PLIC (FC and FDC) and SCR (FDC). The highest effect sizes in the brain (e.g. −1.11 for total cerebellum volume or −1.17 for FC in PLIC) were similar to the highest effect sizes in the spinal cord (−0.95 for CSA at C2–C3, −1.23 for CSA at C1–C2). We also note that the longitudinal effect sizes are not exactly comparable between our brain results and spinal cord results, because of differences in data analysis (using only same scanner data only for the brain results). However, the 1-year effect sizes (calculated from 1-year follow-up data only and provided in Supplementary Table 6 in this paper and Supplementary Table 7 in the previous paper^20^ on findings in the spinal cord in the same cohort) are directly comparable. Taken together, these results indicate that, although the spinal cord cross-sectional area is particularly sensitive to disease progression (thereby reflecting the well-known hallmark degeneration of dorsal root ganglia in FRDA), morphometric and microstructural brain MRI metrics might also provide sensitive biomarkers of disease progression, particularly in the early stage of the disease. This is further supported by relatively strong (>0.5) and highly significant correlations between clinical metrics and C2–C3 spinal cord area and FDC of the SCP. These findings are also consistent with recent work^12^ demonstrating early and progressive reduction of the SCP (as well as ICP and dentate nucleus) volume, and with the central role of the SCP in sensory, motor and speech impairments (via the cerebellothalamic tract) in FRDA.

The primary limitation of this study is the upgrade to the MR scanner from Trio to Prisma during data collection. Data from five volunteers scanned before and after the upgrade showed no significant difference between Trio and Prisma for any of the MR metrics. However, this comparison did not have sufficient power to rule out more subtle effects of the upgrade. When looking more closely at longitudinal results, we did observe small (on the order of 1%) effects of the scanner upgrade (e.g. in the cerebellum, Supplementary Fig. 1). Hence, for each individual subject, we only used data from the same scanner to rule out any possible bias. Another limitation is that there was no longitudinal data for the control group and thus longitudinal slopes for the FRDA group were evaluated assuming that the control group remained stable over time. This is a reasonable assumption in our cohort (mean age 19 years at baseline), since brain development reaches a plateau in early twenties and remains relatively stable in the twenties and thirties.^54,55^ Furthermore, the relatively small sample size likely limits the power of DTI and FBA metrics to estimate the full extent of microstructural alterations in this population, especially in longitudinal voxel-based analysis (TBSS and FBA) that used only 1-year follow-up data.

In conclusion, we report macrostructural and microstructural alterations and longitudinal changes at early stage in FRDA. We found significant volume changes over time in the cerebellum, brain (thalamus and ventral diencephalon), brainstem (pons and midbrain) and in the fourth ventricle. DTI also showed significant longitudinal changes primarily in SCP (decrease in FA and increase in RD). This is also the first study to use higher order diffusion MRI models (FBA) to demonstrate that microstructural alterations in FRDA are characterized by a reduction in white matter fibre tracts density and volume. We found significant longitudinal changes in FBA metrics in SCP, PLIC and SCR.

Our study shows that several MR metrics are sensitive to disease progression in the brain. The highest longitudinal effect sizes were observed for total cerebellar white matter and total cerebellum volume, even though there was practically no cerebellar atrophy at baseline except in the cerebellar white matter. Diffusion metrics in the SCP (as well as in SCR and PLIC) showed comparable effect sizes. These MR metrics may therefore constitute useful novel biomarkers of disease progression for clinical trials.

## Supplementary Material

fcad196_Supplementary_Data

## Data Availability

The data that support the findings of this study are available upon reasonable request from the corresponding author. The data are not publicly available due to their containing information that could compromise the privacy of research participants.

## References

[1] Pandolfo M . Friedreich ataxia. Arch Neurol. 2008;65(10):1296–12303. 18852343 10.1001/archneur.65.10.1296

[2] Dürr A , Cossee M, Agid Y, et al Clinical and genetic abnormalities in patients with Friedreich’s ataxia. N Engl J Med. 1996;335(16):1169–1175. 8815938 10.1056/NEJM199610173351601

[3] Adanyeguh IM , Perlbarg V, Henry PG, et al Autosomal dominant cerebellar ataxias: Imaging biomarkers with high effect sizes. Neuroimage Clin. 2018;19:858–867. 29922574 10.1016/j.nicl.2018.06.011 PMC6005808

[4] Adanyeguh IM , Lou X, McGovern E, et al Multiparametric in vivo analyses of the brain and spine identify structural and metabolic biomarkers in men with adrenomyeloneuropathy. Neuroimage Clin. 2021;29:102566. 10.1016/j.nicl.2021.102566 PMC7847955 33516063

[5] Della Nave R , Ginestroni A, Giannelli M, et al Brain structural damage in Friedreich’s ataxia. J Neurol Neurosurg Psychiatry. 2008;79(1):82–85. 17634216 10.1136/jnnp.2007.124297

[6] Vavla M , Arrigoni F, Nordio A, et al Functional and structural brain damage in Friedreich’s ataxia. Front Neurol. 2018;9:747. 30237783 10.3389/fneur.2018.00747 PMC6135889

[7] França MC Jr , D’Abreu A, Yasuda CL, et al A combined voxel-based morphometry and 1H-MRS study in patients with Friedreich’s ataxia. J Neurol. 2009;256(7):1114–1120. 19280106 10.1007/s00415-009-5079-5

[8] Rezende TJ , Silva CB, Yassuda CL, et al Longitudinal magnetic resonance imaging study shows progressive pyramidal and callosal damage in Friedreich’s ataxia. Mov Disord. 2016;31(1):70–78. 26688047 10.1002/mds.26436

[9] Lindig T , Bender B, Kumar VJ, et al Pattern of cerebellar atrophy in Friedreich’s ataxia—using the SUIT template. Cerebellum. 2019;18(3):435–447. 30771164 10.1007/s12311-019-1008-z

[10] Selvadurai LP , Corben LA, Delatycki MB, et al Multiple mechanisms underpin cerebral and cerebellar white matter deficits in Friedreich ataxia: The IMAGE-FRDA study. Hum Brain Mapp. 2020;41(7):1920–1933. 31904895 10.1002/hbm.24921 PMC7267947

[11] Selvadurai LP , Georgiou-Karistianis N, Shishegar R, et al Longitudinal structural brain changes in Friedreich ataxia depend on disease severity: The IMAGE-FRDA study. J Neurol. 2021;268(11):4178–4189. 33860369 10.1007/s00415-021-10512-x

[12] Harding IH , Chopra S, Arrigoni F, et al Brain structure and degeneration staging in Friedreich ataxia: Magnetic resonance imaging volumetrics from the ENIGMA-ataxia working group. Ann Neurol. 2021;90(4):570–583. 34435700 10.1002/ana.26200 PMC9292360

[13] Rizzo G , Tonon C, Valentino ML, et al Brain diffusion-weighted imaging in Friedreich’s ataxia. Mov Disord. 2011;26(4):705–712. 21370259 10.1002/mds.23518

[14] Rezende TJR , Martinez ARM, Faber I, et al Structural signature of classical versus late-onset Friedreich’s ataxia by Multimodality brain MRI. Hum Brain Mapp. 2017;38(8):4157–4168. 28543952 10.1002/hbm.23655 PMC6866843

[15] Pagani E , Ginestroni A, Della Nave R, et al Assessment of brain white matter fiber bundle atrophy in patients with Friedreich ataxia. Radiology. 2010;255(3):882–889. 20501725 10.1148/radiol.10091742

[16] Della Nave R , Ginestroni A, Diciotti S, Salvatore E, Soricelli A, Mascalchi M. Axial diffusivity is increased in the degenerating superior cerebellar peduncles of Friedreich’s ataxia. Neuroradiology. 2011;53(5):367–372. 21128070 10.1007/s00234-010-0807-1

[17] Akhlaghi H , Yu J, Corben L, et al Cognitive deficits in Friedreich ataxia correlate with micro-structural changes in dentatorubral tract. Cerebellum. 2014;13(2):187–198. 24085646 10.1007/s12311-013-0525-4

[18] Dogan I , Tinnemann E, Romanzetti S, et al Cognition in Friedreich’s ataxia: A behavioral and multimodal imaging study. Ann Clin Transl Neurol. 2016;3(8):572–587. 27606341 10.1002/acn3.315 PMC4999591

[19] Rezende TJR , Martinez ARM, Faber I, et al Developmental and neurodegenerative damage in Friedreich’s ataxia. Eur J Neurol. 2019;26(3):483–489. 30326180 10.1111/ene.13843

[20] Joers JM , Adanyeguh IM, Deelchand DK, et al Spinal cord magnetic resonance imaging and spectroscopy detect early-stage alterations and disease progression in Friedreich ataxia. Brain Commun. 2022;4(5):fcac246. 10.1093/braincomms/fcac246 PMC9581897 36300142

[21] Mascalchi M , Toschi N, Giannelli M, et al Regional cerebral disease progression in Friedreich’s ataxia: A longitudinal diffusion tensor imaging study. J Neuroimaging. 2016;26(2):197–200. 26175281 10.1111/jon.12270

[22] Mascalchi M , Bianchi A, Ciulli S, et al Lower medulla hypoplasia in Friedreich ataxia: MR imaging confirmation 140 years later. J Neurol. 2017;264(7):1526–1528. 28620720 10.1007/s00415-017-8542-8

[23] Zalesky A , Akhlaghi H, Corben LA, et al Cerebello-cerebral connectivity deficits in Friedreich ataxia. Brain Struct Funct. 2014;219(3):969–981. 23563750 10.1007/s00429-013-0547-1

[24] Jones DK , Knösche TR, Turner R. White matter integrity, fiber count, and other fallacies: The do’s and don’ts of diffusion MRI. Neuroimage. 2013;73:239–254. 22846632 10.1016/j.neuroimage.2012.06.081

[25] Raffelt DA , Tournier JD, Smith RE, et al Investigating white matter fibre density and morphology using fixel-based analysis. Neuroimage. 2017;144(Pt A):58–73. 27639350 10.1016/j.neuroimage.2016.09.029 PMC5182031

[26] Subramony SH , May W, Lynch D, et al Measuring Friedreich ataxia: Interrater reliability of a neurologic rating scale. Neurology. 2005;64(7):1261–1262. 15824358 10.1212/01.WNL.0000156802.15466.79

[27] Schmitz-Hübsch T , du Montcel ST, Baliko L, et al Scale for the assessment and rating of ataxia: Development of a new clinical scale. Neurology. 2006;66(11):1717–1720. 16769946 10.1212/01.wnl.0000219042.60538.92

[28] Reuter M , Schmansky NJ, Rosas HD, Fischl B. Within-subject template estimation for unbiased longitudinal image analysis. Neuroimage. 2012;61(4):1402–1418. 22430496 10.1016/j.neuroimage.2012.02.084 PMC3389460

[29] Iglesias JE , Van Leemput K, Bhatt P, et al Bayesian segmentation of brainstem structures in MRI. NeuroImage. 2015;113:184–195. 25776214 10.1016/j.neuroimage.2015.02.065 PMC4434226

[30] Carass A , Cuzzocreo JL, Han S, et al Comparing fully automated state-of-the-art cerebellum parcellation from magnetic resonance images. Neuroimage. 2018;183:150–172. 30099076 10.1016/j.neuroimage.2018.08.003 PMC6271471

[31] Romero JE , Coupe P, Giraud R, et al CERES: A new cerebellum lobule segmentation method. Neuroimage. 2017;147:916–924. 27833012 10.1016/j.neuroimage.2016.11.003

[32] Veraart J , Novikov DS, Christiaens D, Ades-Aron B, Sijbers J, Fieremans E. Denoising of diffusion MRI using random matrix theory. NeuroImage. 2016;142:394–406. 27523449 10.1016/j.neuroimage.2016.08.016 PMC5159209

[33] Kellner E , Dhital B, Kiselev VG, Reisert M. Gibbs-ringing artifact removal based on local subvoxel-shifts. Magn Reson Med. 2016;76(5):1574–1581. 26745823 10.1002/mrm.26054

[34] Andersson JLR , Graham MS, Zsoldos E, Sotiropoulos SN. Incorporating outlier detection and replacement into a non-parametric framework for movement and distortion correction of diffusion MR images. Neuroimage. 2016;141:556–572. 27393418 10.1016/j.neuroimage.2016.06.058

[35] Andersson JLR , Graham MS, Drobnjak I, Zhang H, Filippini N, Bastiani M. Towards a comprehensive framework for movement and distortion correction of diffusion MR images: Within volume movement. Neuroimage. 2017;152:450–466. 28284799 10.1016/j.neuroimage.2017.02.085 PMC5445723

[36] Andersson JLR , Sotiropoulos SN. An integrated approach to correction for off-resonance effects and subject movement in diffusion MR imaging. NeuroImage. 2016;125:1063–1078. 26481672 10.1016/j.neuroimage.2015.10.019 PMC4692656

[37] Andersson JLR , Skare S, Ashburner J. How to correct susceptibility distortions in spin-echo echo-planar images: Application to diffusion tensor imaging. NeuroImage. 2003;20(2):870–888. 14568458 10.1016/S1053-8119(03)00336-7

[38] Tustison NJ , Avants BB, Cook PA, et al N4ITK: Improved N3 bias correction. IEEE Trans Med Imaging. 2010;29(6):1310–1320. 20378467 10.1109/TMI.2010.2046908 PMC3071855

[39] Dhollander T , Raffelt D, Connelly A. Unsupervised 3-tissue response function estimation from single-shell or multi-shell diffusion MR data without a co-registered T1 image. In: ISMRM Workshop on Breaking the Barriers of Diffusion MRI. 2016

[40] Jeurissen B , Tournier JD, Dhollander T, Connelly A, Sijbers J. Multi-tissue constrained spherical deconvolution for improved analysis of multi-shell diffusion MRI data. NeuroImage. 2014;103:411–426. 25109526 10.1016/j.neuroimage.2014.07.061

[41] Smith RE , Tournier JD, Calamante F, Connelly A. SIFT: Spherical-deconvolution informed filtering of tractograms. Neuroimage. 2013;67:298–312. 23238430 10.1016/j.neuroimage.2012.11.049

[42] Medawar E , Thieleking R, Manuilova I, et al Estimating the effect of a scanner upgrade on measures of grey matter structure for longitudinal designs. PLoS One. 2021;16(10):e0239021. 10.1371/journal.pone.0239021 PMC8491918 34610020

[43] Plitman E , Bussy A, Valiquette V, et al The impact of the Siemens Tim Trio to Prisma upgrade and the addition of volumetric navigators on cortical thickness, structure volume, and (1)H-MRS indices: An MRI reliability study with implications for longitudinal study designs. Neuroimage. 2021;238:118172. 10.1016/j.neuroimage.2021.118172 34082116

[44] Takao H , Hayashi N, Kabasawa H, Ohtomo K. Effect of scanner in longitudinal diffusion tensor imaging studies. Hum Brain Mapp. 2012;33(2):466–477. 21391276 10.1002/hbm.21225 PMC6869949

[45] Smith SM , Jenkinson M, Johansen-Berg H, et al Tract-based spatial statistics: Voxelwise analysis of multi-subject diffusion data. Neuroimage. 2006;31(4):1487–1505. 16624579 10.1016/j.neuroimage.2006.02.024

[46] Keihaninejad S , Zhang H, Ryan NS, et al An unbiased longitudinal analysis framework for tracking white matter changes using diffusion tensor imaging with application to Alzheimer’s disease. Neuroimage. 2013;72:153–163. 23370057 10.1016/j.neuroimage.2013.01.044

[47] Raffelt DA , Smith RE, Ridgway GR, et al Connectivity-based fixel enhancement: Whole-brain statistical analysis of diffusion MRI measures in the presence of crossing fibres. Neuroimage. 2015;117:40–55. 26004503 10.1016/j.neuroimage.2015.05.039 PMC4528070

[48] Ward PGD , Harding IH, Close TG, et al Longitudinal evaluation of iron concentration and atrophy in the dentate nuclei in Friedreich ataxia. Mov Disord. 2019;34(3):335–343. 30624809 10.1002/mds.27606

[49] Friedreich N . Ueber Ataxie mit besonderer Berücksichtigung der hereditären Formen. Archive f Pathol Anat. 1876;68(2):145–245.

[50] Mascalchi M . The cerebellum looks normal in Friedreich ataxia. AJNR Am J Neuroradiol. 2013;34(2):E22. 23328073 10.3174/ajnr.A3480 PMC7965124

[51] Akakin A , Peris-Celda M, Kilic T, Seker A, Gutierrez-Martin A, Rhoton A Jr. The dentate nucleus and its projection system in the human cerebellum: The dentate nucleus microsurgical anatomical study. Neurosurgery. 2014;74(4):401–424. 24448179 10.1227/NEU.0000000000000293

[52] Akhlaghi H , Corben L, Georgiou-Karistianis N, et al Superior cerebellar peduncle atrophy in Friedreich’s ataxia correlates with disease symptoms. Cerebellum. 2011;10(1):81–87. 21107777 10.1007/s12311-010-0232-3

[53] Jeurissen B , Leemans A, Tournier JD, Jones DK, Sijbers J. Investigating the prevalence of complex fiber configurations in white matter tissue with diffusion magnetic resonance imaging. Hum Brain Mapp. 2013;34(11):2747–2766. 22611035 10.1002/hbm.22099 PMC6870534

[54] Behler A , Kassubek J, Müller HP. Age-related alterations in DTI metrics in the human brain—consequences for age correction. Front Aging Neurosci. 2021;13:682109. 10.3389/fnagi.2021.682109 PMC8239142 34211389

[55] Levman J , MacDonald P, Lim AR, Forgeron C, Takahashi E. A pediatric structural MRI analysis of healthy brain development from newborns to young adults. Hum Brain Mapp. 2017;38(12):5931–5942. 28898497 10.1002/hbm.23799 PMC5696794

