# Supplementary material for: Brain MRI detects early-stage alterations and disease progression in Friedreich ataxia
Source: Brain Commun. 2023 Jul 6;5(4):fcad196. doi: 10.1093/braincomms/fcad196 (PMC10360047; doi:10.1093/braincomms/fcad196)
Supplement: fcad196_Supplementary_Data [file fcad196_supplementary_data.pdf]

SUPPLEMENTARY DATA

Table of Contents

Supplementary Figure 1: Effect of scanner upgrade on total volume of the cerebellum.

Supplementary Figure 2: Effects of tensor-based vs. scalar-based diffusion tensor imaging registration.

Supplementary Figure 3: Longitudinal tract-based spatial statistics in FRDA participants

Supplementary Figure 4: Longitudinal voxel-wise analysis of fixel-based analysis metrics in FRDA participants

Supplementary Table 1: Test-retest Trio-Prisma data

Supplementary Table 2: Cross-sectional brain volumetry in controls and patients

Supplementary Table 3: Cross-sectional brain DTI metrics

Supplementary Table 4: Cross-sectional brain FBA metrics

Supplementary Table 5: Longitudinal slopes for all variables

Supplementary Table 6: Effect sizes at 1-year follow-up

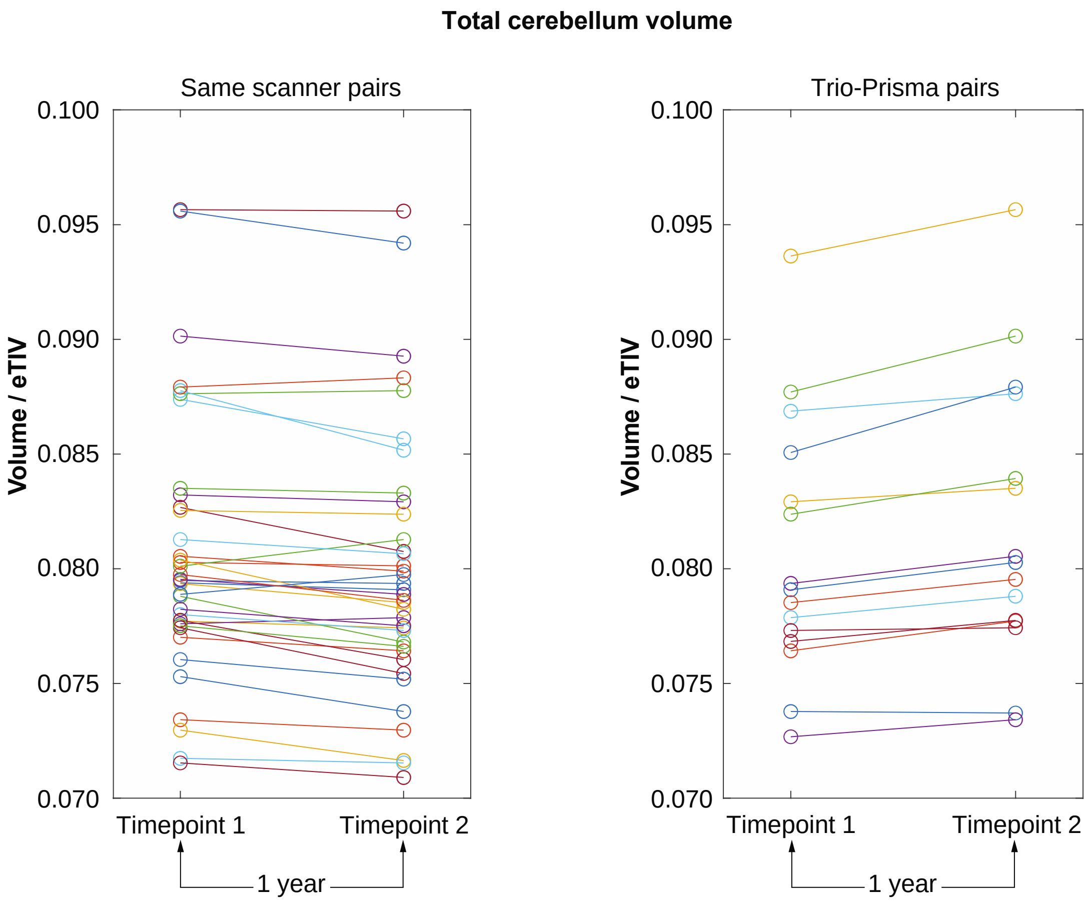

Supplementary Figure 1: Effect of scanner upgrade on total volume of the cerebellum. Same-scanner pairs of values at two consecutive time points (Trio-Trio or Prisma-Prisma) showed mostly a decreasing trend, while most Trio-Prisma pairs (with the upgrade happening in between measurements) showed an increasing trend. The average change was -0.7% per year with same-scanner pairs +1.3% per year for Trio-Prisma pairs and the difference was highly significant. This led us to keep only time points from the same scanner for each individual subject. eTIV = estimated total intracranial volume.

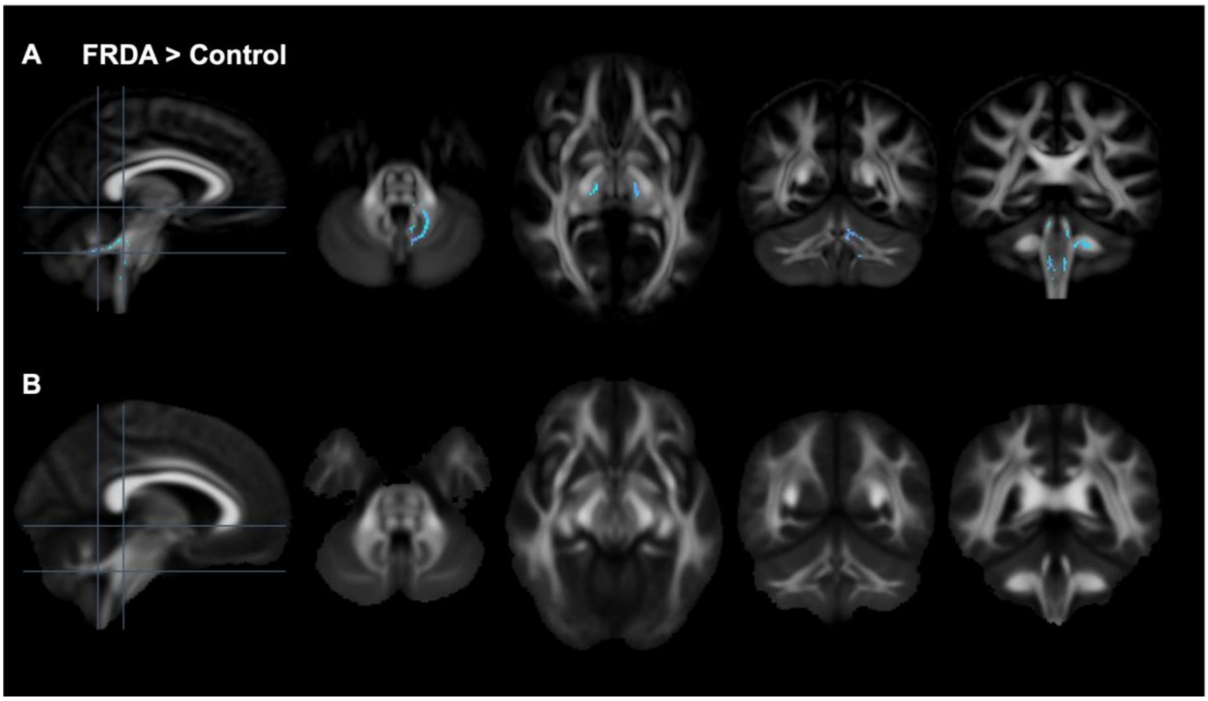

Supplementary Figure 2: Effects of tensor-based vs. scalar-based diffusion tensor imaging registration. A) Differences in axial diffusivity (AD) between control and FRDA participants are observed in the cerebellar peduncles using the tensor-based registration (DTI-TK). B) The standard scalar-based registration shows no differences in AD.

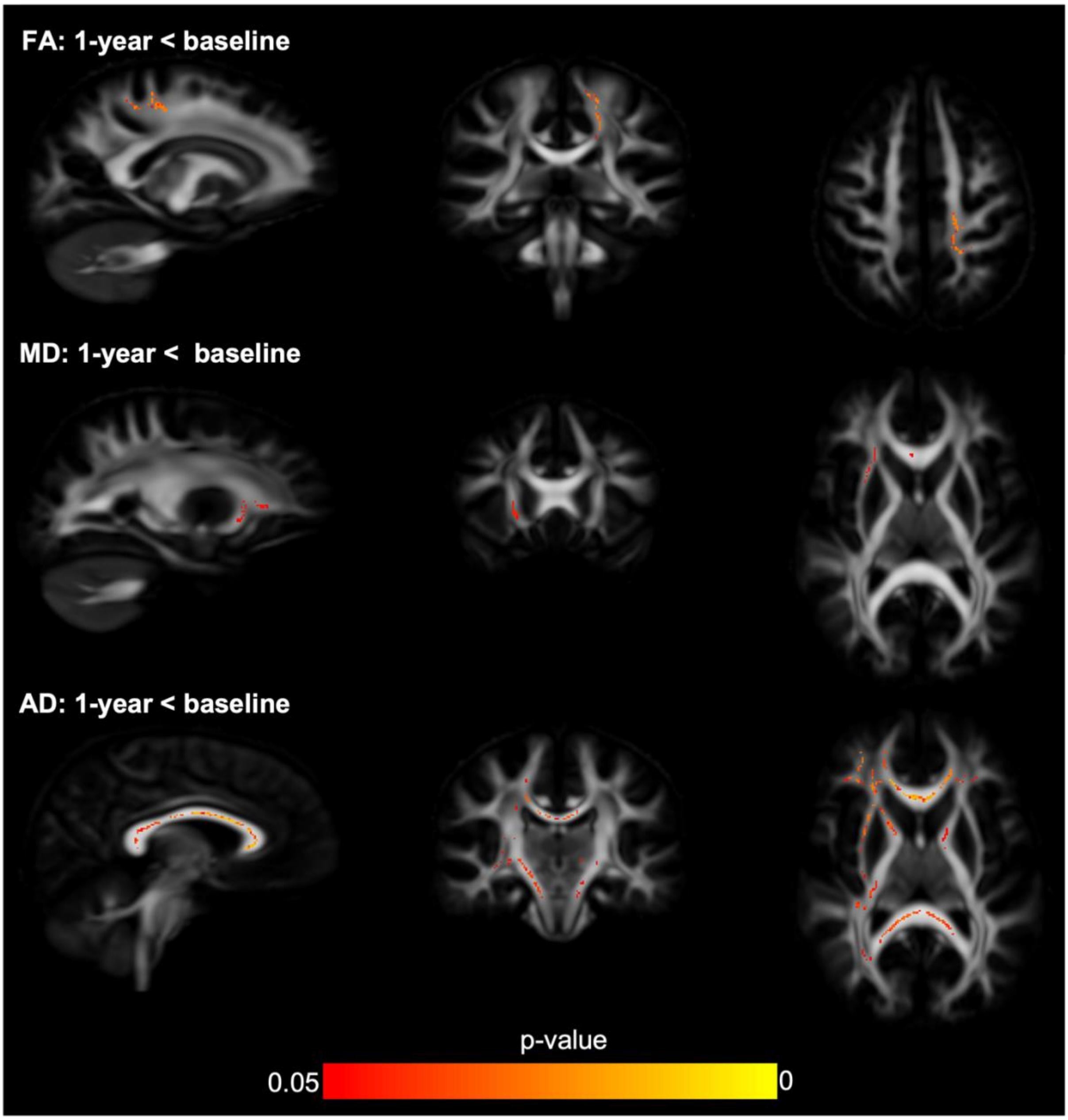

Supplementary Figure 3: Longitudinal tract-based spatial statistics in FRDA participants, comparing baseline and 1-year follow-up for datasets available on the same scanner. Fractional anisotropy (FA) and mean diffusivity (MD) showed a sparse decrease at 1-year follow-up while axial diffusivity (AD) showed decrease in many brain regions.

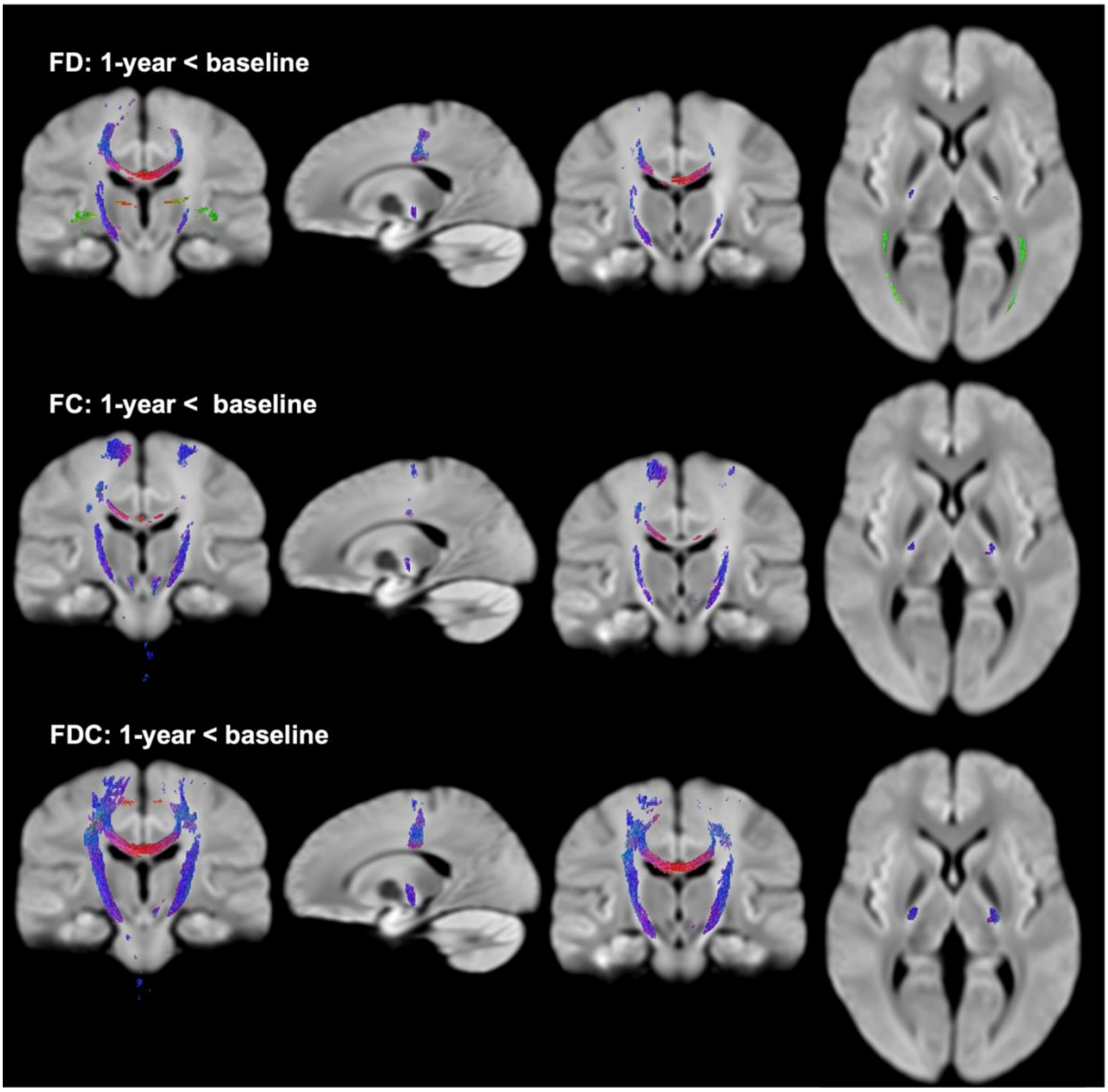

Supplementary Figure 4: Longitudinal voxel-wise analysis of fixel-based analysis metrics in FRDA participants, comparing baseline and 1-year follow-up for datasets available on the same scanner. Fiber density (FD), fiber cross-section (FC), and fiber density and cross-section (FDC) were reduced after 1-year follow-up.

Supplementary Table 1: Test-retest Trio-Prisma data

Metric	Trio	Prisma	Difference	p-value	
			(%)	Raw	Corrected
**Morphometry - FreeSurfer (10^{-2})**					
Medulla	0.481 ± 0.039	0.513 ± 0.015	6.5%	0.190	1
Pons	1.501 ± 0.088	1.530 ± 0.087	2.0%	0.220	1
Midbrain	0.637 ± 0.028	0.617 ± 0.031	-3.3%	**0.017**	0.204
Thalamus	1.489 ± 0.101	1.451 ± 0.102	-2.6%	0.321	1
4th ventricle	0.216 ± 0.030	0.223 ± 0.028	3.6%	0.333	1
SCP	0.027 ± 0.002	0.032 ± 0.004	19.0%	0.055	0.605
CSF	0.105 ± 0.022	0.114 ± 0.031	8.3%	0.126	1
VentralDC	0.792 ± 0.046	0.746 ± 0.077	-5.8%	0.176	1
CC mid-posterior	0.048 ± 0.005	0.048 ± 0.007	1.2%	0.721	1
**Morphometry - CERES (10^{-2})**					
Cerebellum	13.535 ± 0.524	13.688 ± 0.485	1.1%	0.270	1
Cerebellum WM	1.592 ± 0.097	1.607 ± 0.107	0.9%	0.352	1
Cerebellum GM	11.943 ± 0.472	12.081 ± 0.44	1.2%	0.275	1
**DTI - FA**					
SCP	0.609 ± 0.033	0.636 ± 0.049	4.4%	0.082	0.41
ICP	0.497 ± 0.009	0.479 ± 0.047	-3.6%	0.447	1
PLIC	0.657 ± 0.019	0.659 ± 0.017	0.2%	0.754	1
SCR	0.468 ± 0.013	0.465 ± 0.013	-0.6%	0.157	0.628
Fx_ST	0.594 ± 0.013	0.592 ± 0.024	-0.4%	0.780	1
**DTI - RD (10^{-3} mm^2/s)**					
SCP	0.505 ± 0.030	0.467 ± 0.058	-7.7%	0.088	0.44
ICP	0.482 ± 0.020	0.502 ± 0.047	4.1%	0.473	1
PLIC	0.348 ± 0.016	0.345 ± 0.009	-0.8%	0.652	1
SCR	0.455 ± 0.007	0.456 ± 0.008	0.1%	0.520	1
Fx_ST	0.454 ± 0.012	0.460 ± 0.024	1.3%	0.513	1
**DTI - MD (10^{-3} mm^2/s)**					
SCP	0.838 ± 0.014	0.805 ± 0.04	-3.9%	0.106	0.424
ICP	0.701 ± 0.022	0.711 ± 0.027	1.4%	0.614	1
PLIC	0.626 ± 0.020	0.624 ± 0.009	-0.3%	0.744	1
SCR	0.625 ± 0.011	0.627 ± 0.011	0.3%	**0.015**	0.075
Fx_ST	0.742 ± 0.005	0.749 ± 0.018	1.0%	0.313	0.939
**DTI - AD (10^{-2} mm^2/s)**					
SCP	0.150 ± 0.005	0.148 ± 0.005	-1.3%	0.329	0.329
ICP	0.114 ± 0.003	0.113 ± 0.002	-0.8%	0.603	0.603
PLIC	0.118 ± 0.005	0.118 ± 0.004	0.0%	0.970	0.970
SCR	0.096 ± 0.003	0.097 ± 0.003	0.5%	**0.043**	**0.043**
Fx_ST	0.132 ± 0.002	0.133 ± 0.003	0.9%	0.266	0.266

Data are presented as mean ± standard deviation

Supplementary Table 2: Cross-sectional brain volumetry in controls and patients

Metric (10^{-2})	Control	FRDA	Difference (%)	Cohen's *d*	p-value Raw	Corrected
**FreeSurfer**						
Medulla	0.322 ± 0.031	0.256 ± 0.018	-20.5%	-2.77	**$4.02x10^{-12}$**	**$1.01x10^{-10}$**
SCP	0.015 ± 0.002	0.011 ± 0.001	-25.1%	-2.00	**$2.36x10^{-08}$**	**$5.66x10^{-07}$**
Midbrain	0.409 ± 0.032	0.370 ± 0.017	-9.6%	-1.62	**$1.87x10^{-06}$**	**$4.31x10^{-05}$**
Pons	0.910 ± 0.080	0.830 ± 0.076	-8.8%	-1.03	**0.001**	**0.023**
Thalamus	1.020 ± 0.064	0.960 ± 0.057	-5.9%	-1.01	**0.001**	**0.029**
CSF	0.110 ± 0.012	0.123 ± 0.022	12.2%	0.73	**0.017**	0.335
VentralDC	0.532 ± 0.038	0.507 ± 0.033	-4.7%	-0.72	**0.019**	0.355
4th ventricle	0.135 ± 0.028	0.159 ± 0.040	17.6%	0.67	**0.027**	0.483
CC mid-posterior	0.029 ± 0.007	0.025 ± 0.005	-12.7%	-0.64	**0.036**	0.606
3rd ventricle	0.057 ± 0.010	0.064 ± 0.015	11.1%	0.49	0.104	1.000
Lateral ventricle	0.605 ± 0.241	0.756 ± 0.425	25.0%	0.42	0.161	1.000
Cerebellum WM	1.842 ± 0.297	1.733 ± 0.254	-5.9%	-0.40	0.184	1.000
Total GM	45.965 ± 3.363	44.741 ± 2.946	-2.7%	-0.39	0.191	1.000
Cerebral WM	28.584 ± 3.339	27.804 ± 1.423	-2.7%	-0.32	0.281	1.000
CC posterior	0.057 ± 0.013	0.055 ± 0.008	-4.9%	-0.28	0.357	1.000
Subcortical GM	3.912 ± 0.326	3.849 ± 0.218	-1.6%	-0.24	0.426	1.000
Caudate	0.493 ± 0.063	0.503 ± 0.062	2.1%	0.17	0.575	1.000
CC	0.197 ± 0.039	0.192 ± 0.026	-2.6%	-0.16	0.587	1.000
CC mid-anterior	0.029 ± 0.006	0.030 ± 0.006	3.2%	0.15	0.623	1.000
Pallidum	0.255 ± 0.029	0.253 ± 0.018	-0.9%	-0.09	0.752	1.000
Amygdala	0.214 ± 0.031	0.212 ± 0.017	-1.0%	-0.09	0.757	1.000
CC anterior	0.052 ± 0.009	0.053 ± 0.008	1.5%	0.09	0.760	1.000
CC central	0.029 ± 0.006	0.029 ± 0.006	-1.4%	-0.07	0.819	1.000
Putamen	0.687 ± 0.086	0.69 ± 0.0540	0.5%	0.05	0.875	1.000
Cerebellum GM	8.291 ± 0.651	8.294 ± 0.586	0.0%	0.00	0.987	1.000
**CERES**						
Cerebellum WM	0.938 ± 0.101	0.842 ± 0.084	-10.3%	-1.05	**$3.0x10^{-04}$**	**0.005**
Lobule III	0.083 ± 0.015	0.076 ± 0.014	-8.0%	-0.66	0.118	1.000
Lobule IV	0.294 ± 0.042	0.271 ± 0.034	-7.7%	-0.58	**0.039**	0.550
Lobule VIIIb	0.548 ± 0.063	0.523 ± 0.064	-4.5%	-0.41	0.176	1.000
Lobule VIIIa	0.825 ± 0.108	0.800 ± 0.078	-3.1%	-0.37	0.373	1.000
Lobule Crus II	1.039 ± 0.176	1.065 ± 0.165	2.6%	0.37	0.619	1.000
Lobule X	0.078 ± 0.012	0.074 ± 0.008	-5.1%	-0.36	0.177	1.000
Cerebellum	8.278 ± 0.793	8.098 ± 0.563	-2.2%	-0.28	0.311	1.000
Lobule I-II	0.007 ± 0.002	0.007 ± 0.002	-1.0%	-0.23	0.988	1.000
Lobule V	0.508 ± 0.095	0.493 ± 0.076	-3.0%	-0.23	0.533	1.000
Lobule IX	0.504 ± 0.094	0.490 ± 0.089	-2.8%	-0.17	0.540	1.000
Lobule VIIb	0.621 ± 0.117	0.623 ± 0.075	0.2%	0.15	0.920	1.000
Cerebellum GM	7.339 ± 0.701	7.256 ± 0.512	-1.1%	-0.15	0.580	1.000
Lobule VI	1.150 ± 0.170	1.121 ± 0.122	-2.5%	-0.11	0.552	1.000
Lobule Crus I	1.683 ± 0.228	1.714 ± 0.211	1.8%	-0.05	0.685	1.000

Data are presented as mean ± standard deviation and are ordered by decreasing absolute value of Cohen's *d*. Volumes are normalized by intracranial volume.

Supplementary Table 3: Cross-sectional brain DTI metrics

Metric	Control	FRDA	Difference (%)	Cohen's *d*	p-value Raw	p-value Corrected
**FA**						
SCP	0.559 ± 0.045	0.437 ± 0.055	-21.8%	-2.38	**2.64×10^{-10}**	**7.14×10^{-9}**
ICP	0.390 ± 0.031	0.313 ± 0.038	-19.7%	-2.20	**2.02×10^{-9}**	**5.24×10^{-8}**
Fx_ST	0.546 ± 0.028	0.517 ± 0.021	-5.4%	-1.21	**1.73×10^{-4}**	**0.004**
ILF_IFOF	0.512 ± 0.026	0.484 ± 0.025	-5.5%	-1.09	**5.85×10^{-4}**	**0.014**
SCR	0.457 ± 0.016	0.437 ± 0.020	-4.4%	-1.07	**7.21×10^{-4}**	**0.017**
PLIC	0.623 ± 0.014	0.604 ± 0.022	-3.2%	-1.02	**0.001**	**0.024**
CP	0.619 ± 0.032	0.592 ± 0.035	-4.4%	-0.80	**0.009**	0.191
SFOF	0.507 ± 0.031	0.480 ± 0.037	-5.2%	-0.77	**0.013**	0.253
PTR	0.552 ± 0.028	0.533 ± 0.026	-3.6%	-0.74	**0.015**	0.291
Tap	0.331 ± 0.051	0.292 ± 0.055	-11.6%	-0.72	**0.018**	0.329
sCC	0.745 ± 0.021	0.732 ± 0.020	-1.8%	-0.66	**0.030**	0.511
RLIC	0.58 ± 0.0170	0.566 ± 0.026	-2.5%	-0.63	**0.038**	0.610
PCR	0.445 ± 0.022	0.433 ± 0.018	-2.7%	-0.62	**0.043**	0.640
bCC	0.693 ± 0.024	0.681 ± 0.022	-1.8%	-0.54	0.076	1
PCT	0.352 ± 0.058	0.326 ± 0.041	-7.3%	-0.53	0.081	1
mLEM	0.455 ± 0.081	0.418 ± 0.071	-8.1%	-0.49	0.104	1
CST	0.376 ± 0.044	0.355 ± 0.047	-5.4%	-0.45	0.137	1
Fx	0.484 ± 0.038	0.459 ± 0.074	-5.2%	-0.41	0.171	1
Cing_h	0.419 ± 0.036	0.408 ± 0.042	-2.6%	-0.27	0.358	1
ACR	0.433 ± 0.026	0.426 ± 0.028	-1.5%	-0.23	0.430	1
SLF	0.457 ± 0.028	0.451 ± 0.029	-1.3%	-0.21	0.479	1
MCP	0.426 ± 0.021	0.421 ± 0.028	-1.2%	-0.21	0.485	1
gCC	0.571 ± 0.028	0.566 ± 0.033	-0.9%	-0.17	0.564	1
ALIC	0.559 ± 0.028	0.554 ± 0.029	-0.8%	-0.17	0.572	1
UNC	0.456 ± 0.039	0.462 ± 0.037	1.3%	0.15	0.609	1
Cing	0.511 ± 0.049	0.515 ± 0.039	0.8%	0.09	0.753	1
EC	0.397 ± 0.023	0.395 ± 0.019	-0.5%	-0.09	0.757	1
**RD (10^{-3} mm^2/s)**						
SCP	0.595 ± 0.064	0.789 ± 0.090	32.6%	2.42	**1.59×10^{-10}**	**4.29×10^{-9}**
ICP	0.821 ± 0.048	0.967 ± 0.121	17.7%	1.49	**7.12×10^{-6}**	**1.85×10^{-4}**
Fx_ST	0.521 ± 0.038	0.561 ± 0.028	7.6%	1.21	**1.77×10^{-4}**	**0.004**
PLIC	0.370 ± 0.014	0.394 ± 0.026	6.5%	1.10	**5.05×10^{-4}**	**0.012**
PTR	0.494 ± 0.041	0.528 ± 0.030	6.8%	0.95	**0.002**	0.057
SCR	0.475 ± 0.022	0.496 ± 0.024	4.5%	0.91	**0.003**	0.073
ILF_IFOF	0.533 ± 0.040	0.566 ± 0.040	6.1%	0.82	**0.008**	0.171
RLIC	0.430 ± 0.018	0.452 ± 0.032	4.9%	0.78	**0.011**	0.210
sCC	0.310 ± 0.026	0.329 ± 0.023	6.0%	0.76	**0.013**	0.247
SFOF	0.438 ± 0.023	0.459 ± 0.031	4.8%	0.75	**0.014**	0.259
Fx	0.788 ± 0.076	0.888 ± 0.178	12.6%	0.68	**0.024**	0.409
Tap	1.253 ± 0.221	1.415 ± 0.258	12.9%	0.67	**0.029**	0.461
CP	0.413 ± 0.041	0.445 ± 0.056	7.8%	0.64	**0.034**	0.510
MCP	0.538 ± 0.021	0.555 ± 0.032	3.2%	0.61	**0.045**	0.629
bCC	0.371 ± 0.030	0.389 ± 0.030	4.7%	0.58	0.056	0.729
PCR	0.534 ± 0.033	0.551 ± 0.028	3.1%	0.54	0.075	0.903
mLEM	0.533 ± 0.103	0.568 ± 0.091	6.6%	0.37	0.221	1
SLF	0.480 ± 0.032	0.491 ± 0.034	2.4%	0.35	0.243	1
ACR	0.519 ± 0.033	0.528 ± 0.034	1.8%	0.28	0.346	1
Cing_h	0.556 ± 0.037	0.566 ± 0.042	1.8%	0.25	0.394	1
ALIC	0.421 ± 0.027	0.428 ± 0.028	1.6%	0.25	0.404	1
EC	0.534 ± 0.025	0.539 ± 0.019	1.0%	0.24	0.418	1
gCC	0.588 ± 0.069	0.601 ± 0.076	2.2%	0.18	0.548	1
CST	0.681 ± 0.079	0.695 ± 0.078	2.0%	0.18	0.551	1
Cing	0.472 ± 0.038	0.476 ± 0.037	0.8%	0.10	0.729	1

Supplementary Table 3 continued

Metric	Control	FRDA	Difference	Cohen's	p-value	
			(%)	*d*	Raw	Corrected
**RD (10^{-3} mm^2/s)**						
PCT	0.595 ± 0.105	0.591 ± 0.076	-0.7%	-0.05	0.880	1
UNC	0.520 ± 0.032	0.520 ± 0.031	-0.1%	-0.02	0.955	1
**MD (10^{-3} mm^2/s)**						
SCP	0.913 ± 0.052	1.053 ± 0.072	15.3%	2.17	**2.93x10^{-9}**	**7.91x10^{-8}**
ICP	1.020 ± 0.046	1.130 ± 0.115	10.7%	1.17	**2.32x10^{-4}**	**0.006**
PLIC	0.636 ± 0.014	0.658 ± 0.022	3.4%	1.15	**3.10x10^{-4}**	**0.008**
Fx_ST	0.790 ± 0.033	0.823 ± 0.030	4.2%	1.06	**8.05x10^{-4}**	**0.019**
RLIC	0.686 ± 0.019	0.706 ± 0.027	3.0%	0.84	**0.007**	0.152
PTR	0.762 ± 0.040	0.791 ± 0.031	3.7%	0.81	**0.009**	0.200
Fx	1.112 ± 0.065	1.207 ± 0.148	8.5%	0.78	**0.011**	0.224
SCR	0.646 ± 0.024	0.663 ± 0.023	2.6%	0.73	**0.017**	0.347
MCP	0.711 ± 0.021	0.730 ± 0.030	2.6%	0.68	**0.025**	0.474
Tap	1.506 ± 0.212	1.650 ± 0.239	9.6%	0.63	**0.037**	0.665
sCC	0.691 ± 0.027	0.705 ± 0.023	2.0%	0.56	0.065	1
SFOF	0.640 ± 0.020	0.652 ± 0.025	1.9%	0.53	0.081	1
ILF_IFOF	0.774 ± 0.040	0.795 ± 0.042	2.8%	0.52	0.082	1
bCC	0.738 ± 0.031	0.754 ± 0.029	2.1%	0.51	0.093	1
CP	0.708 ± 0.037	0.730 ± 0.048	3.0%	0.49	0.101	1
PCR	0.718 ± 0.036	0.733 ± 0.031	2.2%	0.48	0.110	1
SLF	0.654 ± 0.026	0.667 ± 0.029	2.0%	0.48	0.111	1
Cing	0.702 ± 0.024	0.712 ± 0.025	1.4%	0.41	0.168	1
ALIC	0.658 ± 0.021	0.665 ± 0.020	1.2%	0.37	0.218	1
EC	0.698 ± 0.020	0.705 ± 0.018	0.9%	0.33	0.271	1
ACR	0.698 ± 0.035	0.706 ± 0.031	1.1%	0.25	0.410	1
mLEM	0.728 ± 0.074	0.745 ± 0.066	2.3%	0.24	0.422	1
PCT	0.734 ± 0.094	0.715 ± 0.070	-2.5%	-0.23	0.438	1
Cing_h	0.743 ± 0.029	0.749 ± 0.032	0.8%	0.20	0.499	1
gCC	0.905 ± 0.070	0.919 ± 0.074	1.6%	0.20	0.503	1
UNC	0.724 ± 0.020	0.728 ± 0.026	0.6%	0.17	0.566	1
CST	0.843 ± 0.074	0.841 ± 0.066	-0.3%	-0.03	0.916	1
**AD (10^{-2} mm^2/s)**						
Fx	0.176 ± 0.006	0.184 ± 0.010	4.8%	0.93	**0.003**	0.072
PLIC	0.117 ± 0.002	0.119 ± 0.003	1.4%	0.64	**0.034**	0.891
PCT	0.101 ± 0.008	0.096 ± 0.007	-4.7%	-0.64	**0.036**	0.899
MCP	0.106 ± 0.003	0.108 ± 0.004	1.9%	0.55	0.069	1
CST	0.117 ± 0.007	0.113 ± 0.006	-2.9%	-0.54	0.072	1
Tap	0.201 ± 0.020	0.212 ± 0.021	5.5%	0.54	0.073	1
RLIC	0.120 ± 0.004	0.122 ± 0.003	1.6%	0.53	0.077	1
SLF	0.100 ± 0.003	0.102 ± 0.003	1.6%	0.53	0.080	1
Cing	0.116 ± 0.005	0.118 ± 0.003	1.9%	0.53	0.083	1
SCP	0.155 ± 0.006	0.158 ± 0.007	2.1%	0.51	0.090	1
Fx_ST	0.133 ± 0.004	0.135 ± 0.005	1.6%	0.45	0.137	1
mLEM	0.112 ± 0.005	0.110 ± 0.005	-1.8%	-0.42	0.157	1
ICP	0.142 ± 0.006	0.146 ± 0.011	2.7%	0.41	0.172	1
ALIC	0.113 ± 0.003	0.114 ± 0.002	0.8%	0.35	0.238	1
PTR	0.130 ± 0.006	0.132 ± 0.005	1.4%	0.34	0.255	1
EC	0.103 ± 0.002	0.104 ± 0.003	0.8%	0.33	0.264	1
PCR	0.108 ± 0.005	0.110 ± 0.004	1.3%	0.31	0.293	1
UNC	0.113 ± 0.003	0.115 ± 0.005	1.2%	0.30	0.306	1
SCR	0.099 ± 0.004	0.100 ± 0.003	0.9%	0.27	0.372	1
bCC	0.147 ± 0.005	0.148 ± 0.004	0.7%	0.23	0.440	1
gCC	0.154 ± 0.008	0.156 ± 0.008	1.1%	0.21	0.485	1

Supplementary Table 3 continued

Metric	Control	FRDA	Difference (%)	Cohen's *d*	p-value	
					Raw	Corrected
**AD (10^{-2} mm²/s)**						
SFOF	0.104 ± 0.004	0.104 ± 0.004	-0.5%	-0.14	0.628	1
ACR	0.106 ± 0.005	0.106 ± 0.004	0.5%	0.12	0.688	1
sCC	0.145 ± 0.004	0.146 ± 0.005	0.3%	0.09	0.751	1
Cing_h	0.112 ± 0.004	0.112 ± 0.004	-0.2%	-0.05	0.876	1
ILF_IFOF	0.126 ± 0.005	0.125 ± 0.006	-0.1%	-0.02	0.957	1
CP	0.130 ± 0.005	0.130 ± 0.004	0.0%	0.01	0.978	1

Data are presented as mean ± standard deviation and are ordered by decreasing absolute value of Cohen's *d*.

Supplementary Table 4: Cross-sectional brain FBA metrics

Metric	Control	FRDA	Difference (%)	Cohen's *d*	p-value Raw	p-value Corrected
**FD**						
SCP	0.889 ± 0.054	0.711 ± 0.066	-20.0%	-2.89	**$9.16x10^{-13}$**	**$7.23x10^{-11}$**
SCR	0.833 ± 0.031	0.780 ± 0.030	-6.3%	-1.73	**$4.89x10^{-7}$**	**$3.57x10^{-5}$**
ICP	0.743 ± 0.045	0.677 ± 0.049	-9.0%	-1.41	**$1.96x10^{-5}$**	**0.001**
CST	0.734 ± 0.055	0.660 ± 0.062	-10.0%	-1.24	**$1.26x10^{-4}$**	**0.008**
CP	0.959 ± 0.048	0.907 ± 0.045	-5.4%	-1.12	**$4.44x10^{-4}$**	**0.029**
PLIC	1.030 ± 0.030	0.994 ± 0.034	-3.4%	-1.10	**$5.45x10^{-4}$**	**0.035**
PCT	0.580 ± 0.063	0.531 ± 0.065	-8.5%	-0.77	**0.013**	0.748
Cing	0.615 ± 0.035	0.636 ± 0.023	3.4%	0.74	**0.016**	0.916
sCC	0.911 ± 0.043	0.888 ± 0.032	-2.5%	-0.62	**0.042**	1
Tap	0.786 ± 0.093	0.737 ± 0.081	-6.2%	-0.57	0.062	1
mLEM	0.747 ± 0.086	0.704 ± 0.083	-5.7%	-0.50	0.094	1
PCR	0.703 ± 0.037	0.688 ± 0.028	-2.2%	-0.49	0.106	1
Fx_ST	0.699 ± 0.031	0.685 ± 0.039	-2.1%	-0.41	0.171	1
Cing_h	0.624 ± 0.045	0.606 ± 0.049	-2.9%	-0.38	0.201	1
bCC	0.928 ± 0.045	0.912 ± 0.042	-1.7%	-0.37	0.216	1
PTR	0.716 ± 0.038	0.704 ± 0.029	-1.7%	-0.37	0.221	1
RLIC	0.827 ± 0.033	0.815 ± 0.043	-1.4%	-0.30	0.313	1
ILF_IFOF	0.655 ± 0.033	0.646 ± 0.030	-1.4%	-0.29	0.326	1
Fx	0.725 ± 0.045	0.705 ± 0.079	-2.7%	-0.29	0.327	1
SFOF	0.700 ± 0.042	0.689 ± 0.033	-1.5%	-0.28	0.345	1
gCC	0.768 ± 0.040	0.776 ± 0.032	1.1%	0.23	0.444	1
UNC	0.778 ± 0.051	0.788 ± 0.069	1.2%	0.16	0.600	1
EC	0.732 ± 0.030	0.736 ± 0.034	0.6%	0.13	0.662	1
ACR	0.619 ± 0.036	0.624 ± 0.040	0.8%	0.13	0.666	1
ALIC	0.718 ± 0.028	0.721 ± 0.030	0.5%	0.12	0.693	1
MCP	0.826 ± 0.046	0.819 ± 0.070	-0.8%	-0.11	0.703	1
SLF	0.695 ± 0.048	0.692 ± 0.036	-0.4%	-0.07	0.812	1
**FC**						
SCP	0.045 ± 0.058	-0.171 ± 0.083	-	-2.93	**$5.92x10^{-13}$**	**$4.74x10^{-11}$**
ICP	0.063 ± 0.062	-0.112 ± 0.070	-	-2.61	**$1.92x10^{-11}$**	**$1.48x10^{-9}$**
mLEM	0.025 ± 0.052	-0.135 ± 0.089	-	-2.12	**$4.96x10^{-9}$**	**$3.77x10^{-7}$**
PLIC	0.014 ± 0.061	-0.081 ± 0.066	-	-1.48	**$8.45x10^{-6}$**	**$6.00x10^{-4}$**
SCR	0.024 ± 0.087	-0.089 ± 0.078	-	-1.38	**$2.58x10^{-5}$**	**0.002**
CP	-0.002 ± 0.082	-0.074 ± 0.086	-	-0.86	**0.005**	0.333
CST	0.034 ± 0.117	-0.049 ± 0.088	-	-0.82	**0.008**	0.482
PTR	0.014 ± 0.058	-0.054 ± 0.116	-	-0.70	**0.021**	1
PCT	0.045 ± 0.089	-0.040 ± 0.166	-	-0.61	**0.043**	1
MCP	0.027 ± 0.091	-0.017 ± 0.111	-	-0.43	0.149	1
PCR	-0.030 ± 0.100	-0.077 ± 0.118	-	-0.43	0.154	1
RLIC	-0.041 ± 0.046	-0.069 ± 0.089	-	-0.37	0.210	1
ILF_IFOF	0.010 ± 0.106	-0.032 ± 0.127	-	-0.35	0.236	1
Fx_ST	-0.044 ± 0.08	-0.075 ± 0.112	-	-0.32	0.288	1
UNC	-0.052 ± 0.097	-0.025 ± 0.100	-	0.28	0.355	1
Fx	-0.044 ± 0.067	-0.017 ± 0.127	-	0.26	0.389	1
SLF	-0.004 ± 0.102	-0.026 ± 0.098	-	-0.22	0.469	1
bCC	-0.022 ± 0.133	-0.047 ± 0.117	-	-0.20	0.497	1
Cing	0.005 ± 0.084	-0.014 ± 0.103	-	-0.19	0.518	1
Tap	-0.076 ± 0.176	-0.042 ± 0.200	-	0.18	0.552	1
SFOF	-0.015 ± 0.088	-0.033 ± 0.125	-	-0.16	0.589	1
ALIC	-0.031 ± 0.109	-0.046 ± 0.115	-	-0.13	0.651	1
ACR	-0.029 ± 0.110	-0.043 ± 0.119	-	-0.12	0.686	1
Cing_h	0.018 ± 0.068	0.010 ± 0.093	-	-0.10	0.744	1

Supplementary Table 4 continued

Metric	Control	FRDA	Difference (%)	Cohen's *d*	p-value Raw	p-value Corrected
**FC**						
sCC	-0.015 ± 0.150	-0.028 ± 0.168	-	-0.08	0.787	1
gCC	-0.055 ± 0.125	-0.055 ± 0.148	-	-0.001	0.997	1
EC	-0.033 ± 0.061	-0.033 ± 0.084	-	0.001	0.998	1
**FDC**						
SCP	0.937 ± 0.093	0.603 ± 0.092	-35.7%	-3.61	**6.54x10^{-16}**	**5.30x10^{-14}**
ICP	0.797 ± 0.077	0.608 ± 0.065	-23.7%	-2.7	**8.45x10^{-12}**	**6.59x10^{-10}**
SCR	0.867 ± 0.097	0.718 ± 0.061	-17.2%	-1.91	**6.41x10^{-8}**	**4.81x10^{-6}**
mLEM	0.765 ± 0.097	0.613 ± 0.071	-19.9%	-1.84	**1.46x10^{-7}**	**1.08x10^{-5}**
PLIC	1.056 ± 0.091	0.920 ± 0.068	-12.9%	-1.73	**5.04x10^{-7}**	**3.63x10^{-5}**
CST	0.774 ± 0.141	0.631 ± 0.06	-18.5%	-1.41	**2.19x10^{-5}**	**0.002**
CP	0.969 ± 0.116	0.847 ± 0.086	-12.6%	-1.23	**0.000**	**0.010**
PCT	0.608 ± 0.079	0.514 ± 0.095	-15.3%	-1.06	**0.001**	0.052
PTR	0.732 ± 0.041	0.676 ± 0.083	-7.7%	-0.82	**0.008**	0.481
PCR	0.689 ± 0.069	0.641 ± 0.067	-7.0%	-0.70	**0.021**	1
RLIC	0.797 ± 0.038	0.765 ± 0.066	-4.1%	-0.57	0.057	1
ILF_IFOF	0.665 ± 0.069	0.632 ± 0.095	-5.0%	-0.39	0.195	1
Fx_ST	0.672 ± 0.063	0.641 ± 0.092	-4.6%	-0.38	0.200	1
MCP	0.861 ± 0.101	0.819 ± 0.123	-4.8%	-0.36	0.223	1
bCC	0.925 ± 0.158	0.880 ± 0.116	-4.9%	-0.34	0.263	1
Cing_h	0.638 ± 0.054	0.617 ± 0.079	-3.4%	-0.31	0.297	1
UNC	0.746 ± 0.107	0.778 ± 0.123	4.4%	0.28	0.350	1
SFOF	0.697 ± 0.083	0.674 ± 0.089	-3.3%	-0.27	0.370	1
sCC	0.914 ± 0.159	0.878 ± 0.148	-3.9%	-0.23	0.433	1
SLF	0.703 ± 0.101	0.685 ± 0.083	-2.6%	-0.20	0.506	1
Cing	0.620 ± 0.058	0.632 ± 0.066	2.0%	0.19	0.513	1
Tap	0.755 ± 0.196	0.730 ± 0.201	-3.3%	-0.13	0.670	1
ALIC	0.704 ± 0.086	0.695 ± 0.080	-1.3%	-0.11	0.701	1
gCC	0.735 ± 0.102	0.746 ± 0.120	1.6%	0.10	0.730	1
EC	0.714 ± 0.058	0.720 ± 0.074	0.8%	0.08	0.774	1
Fx	0.699 ± 0.061	0.694 ± 0.095	-0.7%	-0.06	0.833	1
ACR	0.607 ± 0.062	0.604 ± 0.068	-0.4%	-0.04	0.899	1

Data are presented as mean ± standard deviation and are ordered by decreasing absolute value of Cohen's *d*.

Supplementary Table 5: Longitudinal slopes for all variables

Metric	Mean	SD	Δ from baseline	SRM	Raw p
**Morphometry - FreeSurfer**					
Cerebellum	-0.000726	0.000759	-0.7%	**-0.96**	1.45×10^{-04}
4th ventricle	0.000065	0.000076	3.9%	**0.86**	4.21×10^{-04}
Ventral diencephalon	-0.000046	0.000055	-0.9%	**-0.85**	4.65×10^{-04}
Thalamus	-0.000175	0.000211	-1.9%	**-0.83**	5.61×10^{-04}
Brainstem	-0.000093	0.000121	-0.7%	**-0.77**	0.001
Pons	-0.000053	0.000087	-0.7%	**-0.60**	0.006
Midbrain	-0.000025	0.000041	-0.7%	**-0.60**	0.006
Cerebellum cortex	-0.000532	0.000900	-0.6%	-0.59	0.007
Total gray matter	-0.003853	0.006624	-0.9%	-0.58	0.007
Subcortical gray matter	-0.000403	0.000701	-1.1%	-0.58	0.008
BrainSegVolNotVentSurf	-0.003853	0.007571	-0.5%	-0.51	0.015
BrainSegVolNotVent	-0.003818	0.007573	-0.5%	-0.50	0.016
BrainSegVol	-0.003461	0.007173	-0.5%	-0.48	0.019
Cerebellum white matter	-0.000194	0.000406	-1.2%	-0.48	0.021
Putamen	-0.000089	0.000192	-1.3%	-0.46	0.024
BrainSegVol_to_eTIV	0.000000	0.000000	-0.5%	-0.44	0.029
Inferior lateral ventricle	0.000013	0.000030	3.4%	0.43	0.030
Lateral ventricle	0.000218	0.000502	2.8%	0.43	0.030
Left hemisphere cortex	-0.001529	0.003569	-1.0%	-0.43	0.032
SupraTentorialVolNotVent	-0.003192	0.007615	-0.5%	-0.42	0.035
Caudate	-0.000033	0.000078	-0.6%	-0.42	0.035
Cortex	-0.002965	0.007105	-0.9%	-0.42	0.035
SupraTentorialVolNotVentVox	-0.003157	0.007625	-0.5%	-0.41	0.036
SupraTentorialVol	-0.002946	0.007352	-0.5%	-0.40	0.041
SCP	-0.000002	0.000006	-2.1%	-0.40	0.041
Right hemisphere cortex	-0.001436	0.003682	-0.9%	-0.39	0.045
3rd ventricle	0.000016	0.000042	2.4%	0.37	0.051
CC mid-anterior	0.000006	0.000015	2.0%	0.37	0.054
CC anterior	0.000006	0.000017	1.2%	0.35	0.062
MaskVol	0.004042	0.011688	0.4%	0.35	0.064
CC central	0.000006	0.000017	2.0%	0.32	0.080
CSF	0.000017	0.000057	1.3%	0.30	0.091
Pallidum	-0.000020	0.000069	-0.8%	-0.29	0.099
Medulla	-0.000013	0.000047	-0.5%	-0.28	0.109
Hippocampus	-0.000019	0.000082	-0.4%	-0.24	0.144
Amygdala	-0.000009	0.000040	-0.4%	-0.22	0.159
CC posterior	0.000003	0.000014	0.6%	0.22	0.161
Optic chiasm	0.000009	0.000043	2.0%	0.22	0.167
CC mid-posterior	0.000003	0.000019	1.4%	0.17	0.218
Accumbens area	-0.000006	0.000036	-0.9%	-0.17	0.221
WM hypointensities	-0.000011	0.000071	-3.1%	-0.16	0.233
Vessel	-0.000002	0.000014	-1.8%	-0.13	0.273
Choroid plexus	0.000005	0.000049	0.4%	0.10	0.328
Cerebral white matter	0.000187	0.002295	0.1%	0.08	0.356
5th ventricle	0.000000	0.000002	4.1%	0.07	0.382
eTIV	0.000000	0.000000	0.0%	-0.03	0.452
**Morphometry – CERES**					
Cerebellum white matter	-0.000083	0.000074	-1.0%	**-1.12**	2.53×10^{-05}
Cerebellum	-0.000572	0.000517	-0.7%	**-1.11**	2.87×10^{-05}
Cerebellum gray matter	-0.000490	0.000481	-0.7%	**-1.02**	7.43×10^{-05}
Lobule Crus I	-0.000141	0.000153	-0.9%	**-0.92**	2.08×10^{-04}
Lobule VI	-0.000116	0.000136	-1.0%	**-0.85**	4.35×10^{-04}
Lobule VIIIb	-0.000042	0.000066	-0.8%	**-0.64**	0.004
Lobule V	-0.000040	0.000064	-0.8%	**-0.62**	0.005

Supplementary Table 5 continued

Metric	Mean	SD	Δ from baseline	SRM	Raw p
**Morphometry - CERES**					
Lobule Crus II	-0.000081	0.000135	-0.7%	**-0.60**	0.006
Lobule IX	-0.000033	0.000079	-0.7%	-0.41	0.036
Lobule I-II-III	-0.000009	0.000025	-1.1%	-0.35	0.061
Lobule X	-0.000004	0.000012	-0.5%	-0.33	0.072
Lobule I-II	-0.000002	0.000006	-2.8%	-0.32	0.080
Lobule III	-0.000007	0.000028	-0.9%	-0.24	0.140
Lobule IV	-0.000008	0.000039	-0.3%	-0.22	0.167
Lobule VIIb	-0.000007	0.000118	-0.1%	-0.06	0.389
Lobule VIIIa	-0.000008	0.000164	-0.1%	-0.05	0.413
**DTI - AD**					
ALIC	-0.000007	0.000005	-0.6%	**-1.32**	5.31×10^{-06}
sCC	-0.000008	0.000006	-0.6%	**-1.31**	6.24×10^{-06}
ACR	-0.000006	0.000008	-0.6%	**-0.76**	0.001
bCC	-0.000006	0.000009	-0.4%	**-0.65**	0.005
MCP	0.000010	0.000016	1.0%	**0.63**	0.005
CP	-0.000016	0.000025	-1.2%	**-0.63**	0.005
PLIC	-0.000004	0.000007	-0.4%	**-0.63**	0.005
PTR	-0.000006	0.000010	-0.5%	**-0.63**	0.006
EC	-0.000003	0.000005	-0.3%	**-0.63**	0.006
SLF	-0.000002	0.000004	-0.2%	-0.57	0.010
SCP	0.000012	0.000022	0.7%	0.55	0.012
ILF_IFOF	-0.000005	0.000010	-0.4%	-0.54	0.013
RLIC	-0.000005	0.000008	-0.4%	-0.54	0.013
ICP	0.000047	0.000090	3.3%	0.52	0.016
PCR	-0.000002	0.000005	-0.2%	-0.50	0.019
CST	0.000019	0.000040	1.7%	0.48	0.023
SFOF	-0.000006	0.000014	-0.6%	-0.44	0.031
PCT	-0.000009	0.000023	-1.0%	-0.40	0.046
gCC	-0.000006	0.000016	-0.4%	-0.35	0.068
Tap	0.000016	0.000052	0.7%	0.31	0.092
Fx	0.000009	0.000033	0.5%	0.27	0.121
Cing_h	-0.000004	0.000014	-0.3%	-0.25	0.135
SCR	-0.000001	0.000005	-0.1%	-0.23	0.159
Fx_ST	-0.000005	0.000024	-0.3%	-0.19	0.197
UNC	0.000002	0.000013	0.2%	0.17	0.222
mLEM	-0.000005	0.000030	-0.5%	-0.17	0.226
Cing	-0.000002	0.000010	-0.1%	-0.15	0.250
**DTI - FA**					
PTR	-0.004458	0.003789	-0.8%	**-1.18**	2.22×10^{-05}
Tap	-0.009746	0.012464	-3.4%	**-0.78**	0.001
SCP	-0.007770	0.013606	-1.7%	-0.57	0.010
Cing_h	0.009597	0.017462	2.3%	0.55	0.012
PCT	-0.010447	0.021329	-3.0%	-0.49	0.021
RLIC	-0.002397	0.005235	-0.4%	-0.46	0.027
UNC	0.006625	0.014622	1.4%	0.45	0.028
ILF_IFOF	-0.002350	0.005668	-0.5%	-0.41	0.040
Cing	0.002871	0.007555	0.5%	0.38	0.053
Fx	-0.007410	0.021576	-1.6%	-0.34	0.071
bCC	-0.001627	0.004747	-0.2%	-0.34	0.071
CP	0.009518	0.029052	1.6%	0.33	0.080
PCR	-0.001451	0.004458	-0.3%	-0.33	0.081
MCP	0.002862	0.011062	0.7%	0.26	0.131
gCC	0.002886	0.011423	0.5%	0.25	0.136
ACR	0.001388	0.005599	0.3%	0.25	0.141

Supplementary Table 5 continued

Metric	Mean	SD	Δ from baseline	SRM	Raw p
**DTI - FA**					
PLIC	-0.000784	0.003736	-0.1%	-0.21	0.180
Fx_ST	-0.001470	0.008479	-0.3%	-0.17	0.224
CST	0.002731	0.018987	0.8%	0.14	0.264
EC	0.000690	0.005029	0.2%	0.14	0.273
mLEM	0.002311	0.035044	0.5%	0.07	0.386
SFOF	0.000613	0.016386	0.1%	0.04	0.434
sCC	-0.000244	0.006535	0.0%	-0.04	0.435
ICP	-0.000680	0.019265	-0.2%	-0.04	0.438
SCR	0.000049	0.004100	0.0%	0.01	0.479
ALIC	-0.000045	0.007203	0.0%	-0.01	0.489
SLF	0.000006	0.002840	0.0%	0.00	0.496
**DTI - MD**					
EC	-0.000003	0.000003	-0.4%	**-0.95**	2.07×10^{-04}
SCP	0.000018	0.000019	1.7%	**0.93**	2.78×10^{-04}
ALIC	-0.000004	0.000004	-0.6%	**-0.88**	4.23×10^{-04}
Cing_h	-0.000009	0.000012	-1.2%	**-0.77**	0.001
UNC	-0.000003	0.000005	-0.5%	**-0.70**	0.003
SFOF	-0.000004	0.000006	-0.6%	**-0.69**	0.003
ACR	-0.000005	0.000007	-0.7%	**-0.68**	0.003
sCC	-0.000004	0.000006	-0.5%	**-0.61**	0.007
ICP	0.000046	0.000078	4.1%	0.58	0.009
CST	0.000013	0.000025	1.6%	0.53	0.015
Tap	0.000027	0.000054	1.6%	0.51	0.018
MCP	0.000006	0.000013	0.9%	0.50	0.018
Cing	-0.000003	0.000006	-0.4%	-0.50	0.019
CP	-0.000017	0.000038	-2.4%	-0.45	0.029
PLIC	-0.000002	0.000004	-0.3%	-0.45	0.029
SLF	-0.000001	0.000003	-0.2%	-0.45	0.029
Fx	0.000016	0.000036	1.3%	0.44	0.033
gCC	-0.000004	0.000016	-0.4%	-0.23	0.157
mLEM	-0.000005	0.000021	-0.7%	-0.23	0.158
bCC	-0.000001	0.000006	-0.2%	-0.22	0.164
SCR	-0.000001	0.000004	-0.1%	-0.22	0.171
ILF_IFOF	-0.000001	0.000005	-0.1%	-0.20	0.187
PCR	-0.000001	0.000003	-0.1%	-0.18	0.210
RLIC	-0.000001	0.000006	-0.1%	-0.18	0.219
PCT	0.000002	0.000012	0.2%	0.12	0.296
Fx_ST	-0.000001	0.000013	-0.1%	-0.07	0.374
PTR	0.000000	0.000005	0.0%	-0.04	0.437
**DTI - RD**					
SCP	0.000021	0.000022	2.7%	**0.97**	1.83×10^{-04}
Cing_h	-0.000012	0.000017	-2.2%	**-0.71**	0.003
EC	-0.000002	0.000004	-0.5%	**-0.61**	0.007
ICP	0.000045	0.000074	4.7%	**0.61**	0.007
UNC	-0.000006	0.000011	-1.2%	-0.59	0.008
Tap	0.000033	0.000056	2.3%	0.58	0.009
PTR	0.000003	0.000005	0.5%	0.57	0.010
ACR	-0.000004	0.000007	-0.8%	-0.56	0.011
Cing	-0.000003	0.000007	-0.7%	-0.51	0.017
CST	0.000010	0.000022	1.5%	0.46	0.026
Fx	0.000019	0.000042	2.1%	0.46	0.028
CP	-0.000018	0.000045	-4.2%	-0.39	0.047
ALIC	-0.000002	0.000006	-0.6%	-0.39	0.050
PCT	0.000007	0.000020	1.2%	0.35	0.070

Supplementary Table 5 continued

Metric	Mean	SD	Δ from baseline	SRM	Raw p
**DTI - RD**					
MCP	0.000005	0.000014	0.8%	0.33	0.076
SLF	-0.000001	0.000004	-0.2%	-0.31	0.091
SFOF	-0.000002	0.000010	-0.5%	-0.25	0.136
sCC	-0.000002	0.000008	-0.5%	-0.20	0.197
PLIC	-0.000001	0.000004	-0.2%	-0.19	0.202
gCC	-0.000003	0.000018	-0.5%	-0.16	0.247
bCC	0.000001	0.000006	0.3%	0.15	0.250
ILF_IFOF	0.000001	0.000006	0.2%	0.15	0.253
mLEM	-0.000005	0.000034	-0.9%	-0.14	0.267
SCR	-0.000001	0.000004	-0.1%	-0.13	0.284
RLIC	0.000001	0.000006	0.2%	0.12	0.294
Fx_ST	0.000001	0.000011	0.2%	0.07	0.373
PCR	0.000000	0.000005	0.0%	0.06	0.403
**FBA - FC**					
MCP	-0.015087	0.010419		**-1.45**	1.66×10^{-06}
PLIC	-0.015104	0.012951		**-1.17**	2.46×10^{-05}
EC	-0.007059	0.007067		**-1.00**	1.32×10^{-04}
mLEM	-0.017041	0.017096		**-1.00**	1.35×10^{-04}
ILF_IFOF	-0.008310	0.009076		**-0.92**	3.08×10^{-04}
SCR	-0.019465	0.021621		**-0.90**	3.61×10^{-04}
ACR	0.010729	0.013055		**0.82**	8.04×10^{-04}
SCP	-0.017919	0.021921		**-0.82**	8.40×10^{-04}
PTR	-0.005775	0.007671		**-0.75**	0.002
Fx_ST	-0.005715	0.007703		**-0.74**	0.002
Tap	0.012183	0.017762		**0.69**	0.003
CP	-0.012296	0.019555		**-0.63**	0.006
PCR	0.005696	0.009996		0.57	0.010
Fx	0.009338	0.016424		0.57	0.010
ICP	-0.009043	0.018887		-0.48	0.023
gCC	0.007067	0.015553		0.45	0.028
CST	-0.010404	0.023791		-0.44	0.033
SLF	0.004134	0.010900		0.38	0.053
bCC	-0.004068	0.011687		-0.35	0.068
ALIC	0.004053	0.014703		0.28	0.116
UNC	-0.004830	0.018267		-0.26	0.126
sCC	0.002960	0.014007		0.21	0.178
Cing	0.001891	0.009620		0.20	0.195
Cing_h	-0.002128	0.012559		-0.17	0.229
SFOF	0.001240	0.008823		0.14	0.269
PCT	0.003882	0.032328		0.12	0.299
RLIC	-0.000356	0.008110		-0.04	0.423
**FBA - FD**					
PTR	-0.009155	0.003870	-1.3%	**-2.37**	1.06×10^{-09}
ACR	-0.007698	0.006305	-1.2%	**-1.22**	1.44×10^{-05}
ALIC	-0.008327	0.007615	-1.2%	**-1.09**	5.08×10^{-05}
ILF_IFOF	-0.008754	0.008172	-1.3%	**-1.07**	6.36×10^{-05}
SFOF	-0.006110	0.007082	-0.9%	**-0.86**	5.30×10^{-04}
EC	-0.007439	0.008669	-1.0%	**-0.86**	5.55×10^{-04}
SCR	-0.006384	0.007644	-0.8%	**-0.84**	7.02×10^{-04}
gCC	-0.006723	0.008913	-0.9%	**-0.75**	0.002
PLIC	-0.006910	0.011755	-0.7%	-0.59	0.008
Fx	-0.008327	0.014746	-1.2%	-0.56	0.010
SCP	-0.014312	0.025843	-1.9%	-0.55	0.011
Tap	-0.004933	0.009101	-0.7%	-0.54	0.013

Supplementary Table 5 continued

Metric	Mean	SD	Δ from baseline	SRM	Raw p
**FBA - FD**					
Fx_ST	-0.004614	0.008644	-0.7%	-0.53	0.014
SLF	-0.003039	0.007897	-0.4%	-0.38	0.051
mLEM	-0.014950	0.043215	-2.0%	-0.35	0.069
bCC	-0.003412	0.010572	-0.4%	-0.32	0.083
Cing	-0.001724	0.005486	-0.3%	-0.31	0.088
Cing_h	0.006588	0.023502	1.1%	0.28	0.113
PCR	-0.002076	0.008054	-0.3%	-0.26	0.132
RLIC	-0.002699	0.010829	-0.3%	-0.25	0.139
PCT	-0.008795	0.043109	-1.6%	-0.20	0.187
sCC	-0.001802	0.009623	-0.2%	-0.19	0.206
UNC	0.002871	0.021004	0.4%	0.14	0.274
ICP	0.005337	0.041857	0.8%	0.13	0.288
CST	0.004737	0.040421	0.7%	0.12	0.303
CP	-0.002114	0.024207	-0.2%	-0.09	0.350
MCP	0.000507	0.022508	0.1%	0.02	0.460
**FBA - FDC**					
PTR	-0.013632	0.007589	-1.9%	**-1.80**	7.87×10^{-08}
ILF_IFOF	-0.014452	0.012410	-2.2%	**-1.16**	2.50×10^{-05}
EC	-0.012225	0.011616	-1.7%	**-1.05**	7.67×10^{-05}
SCP	-0.026530	0.025556	-3.7%	**-1.04**	8.88×10^{-05}
SCR	-0.021441	0.021275	-2.9%	**-1.01**	1.21×10^{-04}
PLIC	-0.021052	0.021666	-2.3%	**-0.97**	1.74×10^{-04}
Fx_ST	-0.008574	0.011476	-1.3%	**-0.75**	0.002
SFOF	-0.005491	0.008824	-0.8%	**-0.62**	0.006
mLEM	-0.025629	0.044822	-3.6%	-0.57	0.010
CP	-0.012290	0.021586	-1.5%	-0.57	0.010
MCP	-0.012636	0.023229	-1.5%	-0.54	0.013
ALIC	-0.005733	0.013887	-0.8%	-0.41	0.040
bCC	-0.008456	0.023057	-1.0%	-0.37	0.059
Tap	0.002772	0.009280	0.4%	0.30	0.099
Cing_h	0.005720	0.022019	0.9%	0.26	0.130
RLIC	-0.002866	0.012456	-0.4%	-0.23	0.158
Fx	-0.003800	0.019902	-0.6%	-0.19	0.202
PCT	-0.006107	0.039266	-1.1%	-0.16	0.248
gCC	-0.001921	0.013639	-0.3%	-0.14	0.268
ACR	-0.001191	0.008852	-0.2%	-0.13	0.277
PCR	0.000945	0.008457	0.1%	0.11	0.312
SLF	-0.000950	0.012710	-0.1%	-0.07	0.371
sCC	-0.000965	0.018177	-0.1%	-0.05	0.407
Cing	-0.000285	0.007963	0.0%	-0.04	0.437
CST	-0.001242	0.042474	-0.2%	-0.03	0.449
UNC	-0.000423	0.034745	-0.1%	-0.01	0.479
ICP	-0.000328	0.035886	-0.1%	-0.01	0.484
**Clinical**					
ADL	1.954783	1.324931		**1.48**	7.07×10^{-07}
FARS total neuro	4.930495	4.244949		**1.16**	1.64×10^{-05}
SARA	2.068959	1.809986		**1.14**	1.17×10^{-04}
Functional staging	0.330311	0.328952		**1.00**	8.64×10^{-05}
9HPT non-dominant	2.953038	4.731900		**0.62**	0.005

Data are ordered by decreasing absolute SRM. Values highlighted in grey are those selected for the longitudinal analysis in the main manuscript.

Supplementary Table 6: Effect sizes at 1-year follow-up

Metric	Mean difference	SD	Δ from baseline	SRM	Raw p
**Morphometry - FreeSurfer**					
Medulla	-0.0000142	0.0000639	-0.59%	-0.22	0.181
Pons	-0.0000462	0.0001208	-0.57%	-0.38	0.024
Midbrain	-0.0000252	0.0000551	-0.70%	-0.46	0.008
Thalamus	-0.0001645	0.0002321	-1.80%	-0.71	1.20×10^{-4}
4th ventricle	0.0000572	0.0000873	3.43%	0.66	2.59×10^{-4}
SCP	-0.0000020	0.0000091	-1.76%	-0.22	0.186
CSF	0.0000204	0.0000735	1.51%	0.28	0.095
Ventral diencephalon	-0.0000387	0.0000954	-0.79%	-0.41	0.017
CC mid-posterior	0.0000015	0.0000199	0.61%	0.07	0.651
**Morphometry – CERES**					
Cerebellum	-0.0005655	0.0007015	-0.70%	-0.81	2.02×10^{-5}
Cerebellum white matter	-0.0000814	0.0001269	-0.96%	-0.64	4.00×10^{-4}
Cerebellum gray matter	-0.0004840	0.0006146	-0.67%	-0.79	2.85×10^{-5}
**DTI - FA**					
SCP	-0.0090557	0.0213527	-1.98%	-0.42	0.017
ICP	-0.0027381	0.0232272	-0.87%	-0.12	0.490
PLIC	-0.0010573	0.0061010	-0.17%	-0.17	0.312
SCR	-0.0006570	0.0046457	-0.15%	-0.14	0.409
Fx_ST	-0.0025096	0.0113983	-0.47%	-0.22	0.201
**DTI - RD**					
SCP	0.0000201	0.0000317	2.62%	0.63	0.001
ICP	0.0000390	0.0000809	4.09%	0.48	0.007
PLIC	-0.0000006	0.0000057	-0.15%	-0.10	0.554
SCR	-0.0000001	0.0000058	-0.02%	-0.02	0.923
Fx_ST	0.0000016	0.0000179	0.30%	0.09	0.590
**FBA - FD**					
SCP	-0.0149077	0.0265885	-1.98%	-0.56	0.002
ICP	0.0013577	0.0372320	0.20%	0.04	0.830
PLIC	-0.0087551	0.0145588	-0.90%	-0.60	0.001
SCR	-0.0070951	0.0096089	-0.93%	-0.74	1.11×10^{-4}
**FBA - FC**					
SCP	-0.0167567	0.0233099		-0.72	1.95×10^{-4}
ICP	-0.0075149	0.0169377		-0.44	0.013
PLIC	-0.0154276	0.0153340		-1.01	9.93×10^{-7}
SCR	-0.0179085	0.0240314		-0.75	9.89×10^{-5}
**FBA - FDC**					
SCP	-0.0266413	0.0310708	-3.72%	-0.86	1.85×10^{-5}
ICP	-0.0033156	0.0338455	-0.51%	-0.10	0.566
PLIC	-0.0227283	0.0252716	-2.47%	-0.90	6.58×10^{-6}
SCR	-0.0208339	0.0251510	-2.80%	-0.83	2.3×10^{-5}
**Clinical**					
SARA	1.9817211	1.6282717		1.22	1.07×10^{-6}
FARS total neuro	4.4999864	4.2858266		1.05	1.44×10^{-7}
Functional staging	0.3037515	0.4154483		0.73	6.39×10^{-5}
ADL	1.9182908	1.9165589		1.00	3.70×10^{-7}
9HPT non-dominant	3.3501324	6.5556231		0.51	3.22×10^{-3}
